# Systematic Review of Polyherbal Combinations Used in Metabolic Syndrome

**DOI:** 10.3389/fphar.2021.752926

**Published:** 2021-10-07

**Authors:** Amber Hanif Palla, Faridah Amin, Bilqees Fatima, Arooj Shafiq, Najeeb Ur Rehman, Ikram ul Haq, Anwar-ul-Hassan Gilani

**Affiliations:** ^1^ Department of Biological and Biomedical Sciences, Aga Khan University Hospital, Karachi, Pakistan; ^2^ Family Medicine, Liaquat National Hospital, Karachi, Pakistan; ^3^ Department of Pharmaceutics, Faculty of Pharmacy, Hamdard University, Karachi, Pakistan; ^4^ Department of Bioscience, Salim Habib University, Karachi, Pakistan; ^5^ Department of Pharmacology and Toxicology, College of Pharmacy, Prince Sattam Bin Abdul Aziz University, Al Kharj, Saudi Arabia; ^6^ National Institute of Health, Islamabad, Pakistan; ^7^ Department of Public Health and Nutrition, The University of Haripur, Haripur, Pakistan

**Keywords:** obesity, natural products, clinical trials, animal models, polyherbal

## Abstract

**Background:** Metabolic syndrome (MetS) is a multifactorial disease, whose main stay of prevention and management is life-style modification which is difficult to attain. Combination of herbs have proven more efficacious in multi-targeted diseases, as compared to individual herbs owing to the “effect enhancing and side-effect neutralizing” properties of herbs, which forms the basis of polyherbal therapies This led us to review literature on the efficacy of herbal combinations in MetS.

**Methods:** Electronic search of literature was conducted by using Cinnahl, Pubmed central, Cochrane and Web of Science, whereas, Google scholar was used as secondary search tool. The key words used were “metabolic syndrome, herbal/poly herbal,” metabolic syndrome, clinical trial” and the timings were limited between 2005–2020.

**Results:** After filtering and removing duplications by using PRISMA guidelines, search results were limited to 41 studies, out of which 24 studies were evaluated for combinations used in animal models and 15 in clinical trials related to metabolic syndrome. SPICE and SPIDER models were used to assess the clinical trials, whereas, a checklist and a qualitative and a semi-quantitative questionnaire was formulated to report the findings for animal based studies. Taxonomic classification of Poly herbal combinations used in animal and clinical studies was designed.

**Conclusion:** With this study we have identified the potential polyherbal combinations along with a proposed method to validate animal studies through systematic qualitative and quantitative review. This will help researchers to study various herbal combinations in MetS, in the drug development process and will give a future direction to research on prevention and management of MetS through polyherbal combinations.

## Introduction

Non-communicable diseases (NCDs) account for 71% of the deaths worldwide with rising prevalence in lower- and middle-income countries (Huang, 2009; Robinson et al., 2013). NCDs have been ranked as one of the top ten global threats in 2019 by World Health Organization (Khowaja et al., 2007; Robinson et al., 2013). Metabolic syndrome (MetS) is a type of NCD with worldwide prevalence ranging from less than 10% to as much as 84% (Rhee et al., 2010) with the burden being greater in South Asian countries (Sever et al., 2003; Su and Li, 2011).

MetS is characterized by a cluster of three or more features including hyperglycaemia, hypertriglyceridemia, a low level of high-density lipoprotein cholesterol (HDL-C), blood pressure and central obesity (Bodeker and Kronenberg, 2002; Anderson and Taylor, 2012). A person who has at least three out of five of these characteristics is labelled as MetS patient. The following criteria should be met for MetS (AuH, 1998; Anderson and Taylor, 2012): waist circumference more than 35 and 40 inches in women and men, respectively (central obesity); triglycerides (TGs) 150 mg/dl or greater, HDL-C less than 50 and 40 mg/dl in women and men, respectively, blood pressure (BP) of 130/85 mm Hg or higher, fasting blood glucose (FBG) of 100 mg/dl or greater. Besides the above mentioned abnormalities, underlying initiators of MetS are inflammation, oxidative stress and insulin resistance (Ma et al., 2009; Aziz et al., 2013; Amin et al., 2015a). Together these factors pose a three- and five-fold greater risk for cardiovascular disease (CVD) and type II diabetes mellitus (T2DM) respectively (Zimmermann et al., 2007), along with high mortality rate (Gilani and Rahman, 2005).

MetS has multiple aetiologies and therefore no single drug can be effective in reversing this situation. The main stay of prevention and management of individuals at risk is life-style modification. However, those who have high levels of risk factors are the recipients for pharmacological treatment which is aimed towards individual symptoms’ management (AuH, 1998; Devalaraja et al., 2011; Mohamed, 2014). Multiple drugs including drugs to lower the blood glucose level, TGs, and blood pressure (Robinson et al., 2013) may be needed for a long time resulting in drug related complications, low compliance rate and high cost of care (Khowaja et al., 2007; Huang, 2009). Alternately, some researchers suggest to advocate life-style modification as the first line therapy for prevention of a chronic disease, rather than using pharmacological therapies such as metformin in pre-diabetes (Rhee et al., 2010) and statins in mild to moderate dyslipidemia (Sever et al., 2003). Endorsing only life-style modifications is challenging for the physicians especially among high-risk patients such as in obese patients, since compliance to dietary modification, and physical activity is difficult to attain (Samir et al., 2011). Therefore, it is imperative to explore innovative therapies which are cost-effective and acceptable, with fewer adverse effects, in order to reduce the risk of cardiovascular diseases (CVD) through addressing the risk factors.

According to World Health Organization (WHO), up to 80% of the Asian population relies on complementary and alternative/Traditional medicine (CAM/T) for their primary healthcare, possibly because more than 80% of people in developing nations can barely afford basic medical needs (Su and Li, 2011). Interestingly, almost half of the population in the developed world also uses CAM/T therapies (Bodeker and Kronenberg, 2002). Amongst the most common complementary modalities used by individuals with CVD risk factors are natural products (Anderson and Taylor, 2012) that have evidently contributed in the development of modern medicine for cardiovascular disorders (AuH, 1998). MetS requires multiple factors to be addressed simultaneously, therefore polyherbal combinations can offer a safe and more effective therapeutic option. Research has revealed that the multi-component properties of polyherbal combinations make them suitable for treating complex diseases and offer great potential for exhibiting synergistic actions. Evaluation of literature from individual effects of potential polyherbal combinations paves the path for deriving new combinations.

Synergistic therapeutic actions of polyherbal formulations are possible through underlying mechanisms such as regulation of same or different targets in various pathways hence in combination enhance efficacy, regulation of enzymes and transporters to improve oral drug bioavailability, neutralize adverse effects and overcome drug resistance mechanisms. Synergism is observed when multiple chemical constituents are present in single or in combination of herbs (Amin et al., 2015a), which are potential therapeutic options for various disease targets. This forms the basis of polyherbal therapies (Ma et al., 2009; Aziz et al., 2013) and is considered rational and more efficacious in multi-targeted diseases (Zimmermann et al., 2007). The effect-enhancing and side-effect neutralizing properties of polyherbal combinations (Gilani and Rahman, 2005) prompted us to review the literature on the efficacy of polyherbal combinations in metabolic syndrome, the incidence of which is rising globally. This will help researchers to identify various effective polyherbal combinations in MetS, which may help in the drug development process, as well as provide future direction towards research on prevention and cure of a menace like metabolic syndrome. Although synergistic therapeutic interactions of herbal ingredients have been frequently reported, to the best of our knowledge, none of the reports have offered review of polyherbal formulations in MetS. Individual herb reviews related to MetS were limited to functional foods (Mohamed, 2014) and exotic fruits (Devalaraja et al., 2011). Hence, in this review, we present recent literature reporting herb synergisms and efficacy of various polyherbal formulations in MetS. We have identified the herb to be good if it manages to modulate at least 3 out of 5 MetS criteria.

## METHODS

### Systematic Review Protocol (Search Strategy and Data Sources)

We decided for a qualitative systematic review for which an electronic literature search was carried out to find articles published mainly in the last 15 years (2005–2020).

For this purpose, following databases, and/or search engines were used: Cinnahl, Pubmed central, Cochrane, Web of Science and Scopus. Google scholar was used as secondary search tool.

The key words used were “metabolic syndrome, herbal/polyherbal,” “metabolic syndrome, clinical trial”.

#### Inclusion Criteria

1. Animal model with MetS that are given more than one herb for treatment.
2. Adults diagnosed with MetS (who qualify for 3 of the 5 MetS parameters: obesity, high blood pressure, hypertriglyceridemia, low HDL, high blood sugar (>100–125 mg/dl).
3. Adults >17 years < 74 years.

#### Exclusion Criteria

1. Review article.
2. Effect of individual herbs on MetS
3. Effect of interventions through diet, low caloric, mediterranean diet etc., on MetS.
4. Any MetS model used but not for the purpose of assessing effect on MetS, rather individual aspect such as obesity, non-alcoholic fatty liver disease and non-alcoholic steatohepatitis and polycystic ovary syndrome.

### Data Analysis and Study Design

All the polyherbal formulations were classified taxonomically and then effect of intervention and evaluation of results were done, based on number of MetS criteria met both in animal and/or humans.

Quality of animal-based studies were assessed by using a qualitative scoring system using 8 questions. Maximum score achieved was 8, with yes = score 1 and no = score 0 with following questions:

1. MetS parameters assessed >3 = score 1; ≤3 = score 0
2. MetS parameters met: 3 out of 5 parameters (good Effect) = 1; <3 out of 5 (not so good) = 0.
3. Dosage of herb provided: Yes = 1; No = 0
4. Components and rationale for dosing: yes = 1; no = 0
5. Animal ethical approval: Yes = 1; No = 0
6. Euthanasia protocol mentioned/followed: Yes = 1; No = 0
7. Model validated for MetS: Yes = 1; No = 0
8. Positive control used: Yes = 1; No = 0

For clinical trial we adopted a mixed model for assessing our articles including SPICE (S = setting; P = population; I = intervention/what; C = comparison/controls E = evaluation/with what result) (Booth, 2006; Cleyle and Booth, 2006) and SPIDER (S = Sample P = phenomenon of interest/intervention I = intervention size, D = design, E = evaluation/outcome R = research type; qualitative, quantitative or mixed type). SPIDER methods had added points for assessing both qualitative and quantitative methods (Cooke et al., 2012). Further aspects of quality of clinical trial were assessed based on following aspects with yes = 1; No = 0 according to an adopted guideline for critical appraisal (Alcántara et al., 2011):

1. The study addresses an appropriate and clearly focused question
2. The assignment of subjects to treatment groups is randomized.
3. An adequate concealment method is used
4. The design keeps subjects and investigators ‘blind’ about treatment allocation.
5. The treatment and control groups are similar at the start of the trial.
6. The only difference between groups is the treatment under investigation.
7. All relevant outcomes are measured in a standard, valid and reliable way
8. What percentage of the individuals or clusters recruited into each treatment arm of the study dropped out before the study was completed?
9. All the subjects are analyzed in the groups to which they were randomly allocated (often referred to as intention to treat analysis)
10. Where the study is carried out at more than one site, results are comparable for all sites.

Besides, following questions were also assessed: concentration of the herb provided or not, quality control of the combination assessed or not and chemical classification done or not.

## Results

**FIGURE 1:**
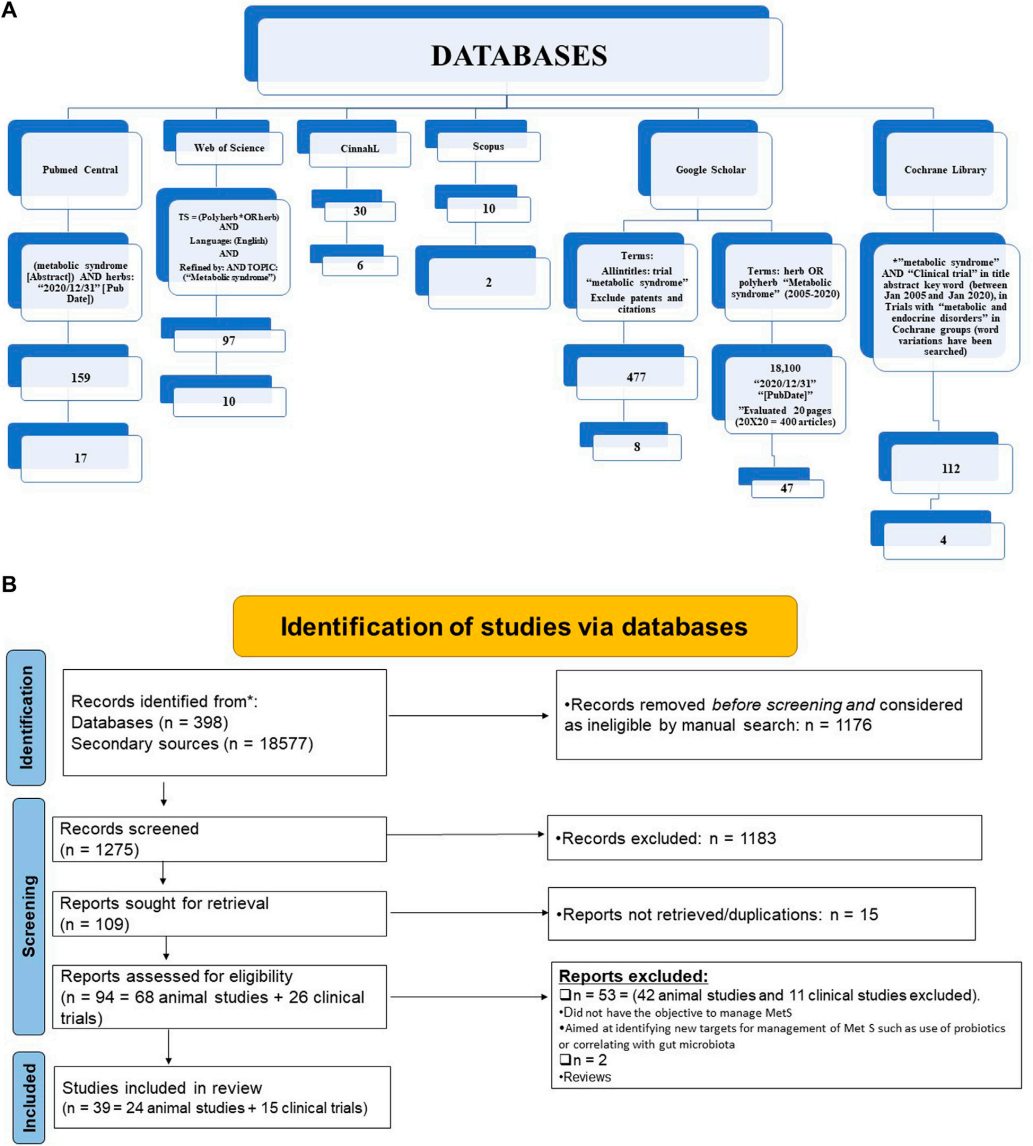
Summary of literature search **(A)** and Analysis of systematic review results according to Prisma guidelines **(B)**.

The selection parameter, applied filters, as well as output of all the searches, are summarised in Figure 1A. In Figure 1B the summary of identified results is presented according to PRISMA guidelines (Page et al., 2019; Maraolo, 2021).

The total reference shortlisted were 109, out of which duplications and or articles which could not be retrieved were removed (*n* = 15) and number of articles to review were 94. Out of total 94 articles, 26 were divided as clinical trials and remaining 68 articles were either based on animal studies or *in-vitro* assays. These articles were further shortlisted by reviewing their basic theme and it was identified that some of the articles did not have the objective to manage MetS or were aimed at identifying new targets for management of Met S such as use of probiotics or correlating with gut microbiota (Ni et al., 2018) or the basic target for those studies were to cater different disease, although parameters for MetS were being met. Hence, out of 68 animal studies, filtered animal studies were identified to be 24 which matched our main objective of MetS. The taxonomic classification of polyherbal combinations used both in animal and clinical studies are summarized in Tables 1, 2, respectively. The meta-analysis of animal studies is summarized in Table 3. To further analyze the quality of studies, a semiquantitative scale was used, the details of which are presented separately as Table 4. The maximum score was 8, and references have been aligned from highest score to lowest score.

**TABLE 1:** Taxonomic classification of all the polyherbal combinations reviewed in animal Studies.

S.No	Reference	Name of the Combination	Components	Chinese Name	Common name	Scientific name	Family	Specie
1	Thota et al. (2014)		*Curcuma longa* (Rhizomes), *Salacia reticulate* (Root), *Gymnema sylvestre* (leaves), *Emblica officinalis* (fruits), *Terminalia chebula* (fruits)		Turmeric	*Curcuma longa* L.	Zingiberaceae	*C. longa*
					Kotala himbatu	*Salacia reticulata*, Wight	Celastraceae	*S*. reticulata
					Gurmar	*Gymnema sylvestre* (Retz.) Schult	Apocynaceae	*G*. sylvestre
					Emblic, myrobalan, Indian gooseberry; Amla	*Emblica officinalis Gaertn*; *Phyllanthus emblica*, *L.*	Phyllanthaceae	*P. emblica*
					Black- or chebulic myrobalan; Haritali	*Terminalia chebula* Retz.	Combretaceae	*T. chebula*
2	Sung et al. (2014)	Dohaekseunggi-tang	*Glycyrrhizae uralensis* Fischer (40 g), *Rheum undulatum* Linne (80 g), *Prunus persica* Linne (60 g), *Cinnamomum cassia* Presl (40 g), and Natrii Sulfas (40 g)	Bo ye da huang	Chinese licorice root; Radix *G*lycyrrhizae	*Glycyrrhiza uralensis*, Fisch. ex DC	Fabaceae	*G. uralensis*
					Rhubarb	*Rheum undulatum* Linne; *Rheum rhabarbarum* L	Polygonaceae	*R. undulatum*; *R. rhubarb arum*
					Peach	*Prunus persica* (L) Batsch	Rosaceae	*P. persica*
					Chinese cinnamon	*Cinnamomum cassia* (L.) J.Presl	Lauraceae	*C. cassia*
					Sodium sulfate (Na_2_SO_4_); main component of mineral Chinese medicine	Natrii sulfas		
3	Li et al. (2013)	Huang-Lian-Jie-Du-Tang	Rhizoma *Coptidis*, Radix *Scutellariae*, Cortex *Phellodendri* and Fructus *Gardeniae* (3:2:2:3)	Huang Lian	Chinese goldthread or canker root	*Coptis chinensis* Franch; *Coptis deltoidea* C.Y. Cheng et Hsiao, and *Coptis teeta* Wall	Ranunculaceae	*C. chinensis; C. deltoidea* and *C. teeta*
				Baikal	Skullcap or Chinese skull cap	*Scutellaria baicalensis*, Georgi	Lamiaceae	*S. baicalensis*
				Huáng bǎi	"Yellow fir" bark of one of two species of Phellodendrn tree: *Phellodendron amurense* or *Phellodendron chinense*	*Phellodendron amurense* Rupr *+ Phellodendron chinense* Schneid	Rutaceae	*P. amurense*, *P. chinense*
					Gardenia; Cape Jasmine	*Gardenia jasminoides*, J.Ellis	Rubiaceae	*G*. *jasminoides*
4	Kho et al. (2016)	RGPM:	Red ginseng and Polygoni Multiflori Radix (1:1)		Red ginseng (produced by steaming and drying fresh and raw ginseng.	Panax ginseng C.A. Meyer	Araliaceae	*P. ginseng*
				Heshuwu	Tuber fleece flower; Chinese climbing knotweed.	*Polygonum multiflorum* Thunb *(Fallopia multiflora* Thunb; *Reynoutria multiflora*	Polygonaceae	*P.multiflorum*, *R. multiflora*
5	Yao et al. (2017b)	Modified lingguizhugan decoction	*Poria cocos* Wolf, *Cinnamomum cassia* Presl, *Atractylodes lancea* DC., *Glycyrrhiza uralensis* Fisch., Nannf. and *Rheum palmatum* L] (ratio of 12:9:6:6:9:9)		Poria cocos, China root	*Wolfiporia cocos* (F.A. Wolf) Ryvarden and Gilb.,	Polyporaceae	*W. extensa*
					Tvach and Guda-tvach; Kirfat-ed-darsini	*Cinnamomum cassia* (L.) Presl	Lauraceae	*C. cassia*
					Southern tsangshu	*Atractylodes lancea* (Thunb) DC	Asteraceae	*A. lancea*
					Glycyrrhizae radix; Liquorice root	*Glycyrrhiza uralensis*, Fisch	Fabaceae	*G. uralensis*
					Chinese rhubarb, Rheum	*Rheum palmatum*	Polygonaceae	*R. palmatum*
6	Amin et al. (2015a)		*Curcuma longa* and *Nigella Sativa*		Turmeric	*Curcuma longa* L.	Zingiberaceae	*C*. *longa*
					Kalonji/black seeds	*Nigella sativa* L.	Ranunculaceae	*N. sativa*
7	Mounts et al. (2015)		soybean meal and probiotics (*Bifidobacterium*, *longum* (BB536)		Mung bean; Soybean meal	*Vigna radiata*, (L.) R. Wilczek, *Testa glycinis*	Fabaceae	*V. radiata*, *T. glycinis*
					Probiotics (BB536)	*Bifidobacterium longum* Reuter 1963	Bifidobacteriaceae	*B. longum*
8	Lee et al. (2015b)	ACE	Artemisia iwayomogi and Curcuma longa (1:1)		Dowijigi	*Artemisia iwayomogi* Kitamura	Compositae/Asteraceae	*A. iwayomogi*
					Turmeric	*Curcuma longa* L.	Zingiberaceae	*C. longa*
9	Hu et al. (2014)	Fu Fang Zhen Zhu Tiao Zhi formula (FTZ)	*Ligustrum lucidum* W.T. Aiton, fructus; *Atractylodes macrocephala* Koid., rhizoma; *Salvia miltiorhiza* Bunge, radix; Coptis chinensis Franch, rhizoma; *Panax noto ginseng* F.H.Chen, radix; Eucommia ulmoides Olive., cortex; *Cirsium japonicum* Fisch. ex DC., radix; *Citrus medica* var. *sarcodactylus Swingle*, fructus		Chinese Privet, Glossy privet	*Ligustrum lucidum*, W.T. Aiton	Oleaceae	*L. lucidum*
					Baizhu (rhizome)	*Atractylodes macrocephala* Koidz.	Compositae/ Asteraceae	*A.macrocephala*
				Danshen	Red sage, Chinese sage		Lamiaceae	*S. miltiorrhiza*
				Huang Lian	Chinese goldthread or canker root	*Salvia miltiorrhiza*, Bunge	Ranunculaceae	*C. chinensis; C. deltoidea* and *C. teeta*
					Sanchi ginseng; Sanqi, Chinese ginseng or notoginseng	*Coptis chinensis* Franch; *Coptis deltoidea* C.Y. Cheng et Hsiao, and *Coptis teeta* Wall	Araliaceae	*P. notoginseng*
					Gutta-Percha	*Panax notoginseng* (Burk) F.H.Chen	Eucommiaceae	*E. ulmoides*
					Japanese thistle	*Eucommia ulmoides* Oliv.	Compositae/Asteraceae	*C. japonicum*
					Fingered citron; Buddha's hand	*Cirsium japonicum* (Thunb) Fisch. ex DC., radix	Rutaceae	*C. medica*
10	Gao et al. (2015)	Erchen decoction	Pericarpium *Citri Reticulatae* (9 g), Rhizoma *Pinelliae* (9 g), *Poria* (6 g) and Radix *Glycyrrhizae* (3 g).		Pericarpium of mandarin orange (dried and ripe peel)	*Citrus medica* var. *sarcodactylus* Swingle	Rutaceae	*C. reticulata*
				Ban Xia	Crowdipper	*Citrus reticulata* *,* *Blanco*	Araceae	*P. ternata*
				Fu ling	Poria cocos, China root	Pinellia ternate (Thunb.) Makino	Polyporaceae	*W. extensa*
					Glycyrrhizae radix; Liquorice root	*Wolfiporia cocos* (F.A. Wolf) Ryvarden and Gilb,	Fabaceae	*G. uralensis*
11	Kaur and C (2012)	CPQ	Curcumin, Piperine and Quercetin in a ratio (94:1:5)		Curcumin (pure chemical from turmeric)	*Glycyrrhiza uralensis*, Fisch	Zingiberaceae	*C. longa*
					Piperine (pure chemical from black pepper	*Curcuma longa* L.	Piperaceae	*P. nigrum*
					Quercetin: Chemical compound (C_15_H_10_O_7_)	*Piper nigrum*, L		
12	Tan et al. (2013)		Extracts of *Salvia miltiorrhiza* and *Gardenia jasminoides*	Danshen	Red sage, Chinese sage	Plant flavonol from the flavonoid group of polyphenols	Lamiaceae	*S. miltiorrhiza*
					Gardenia; Cape Jasmine	*Salvia miltiorrhiza*, Bunge	Rubiaceae	*G. jasminoides*
13	Wei et al. (2012)	SUB885C	*Fructus Crataegi , Folium Nelumbinis, Folium Apocyni*, Flos *Rosaen rugosae* , Radix et Rhizoma Rhei , *Depuratum mirabilitum, Thallus Sargassi*, and honey fried Radix Glycyrrhizae		Single-seeded hawthorn; Hawthorn Berry	*Gardenia jasminoides*, J.Ellis	Rosaceae	*C. monogyna*
				He ye herb	Lotus leaf	Crataegus monogyna Jacq	Nelumbonaceae/Nymphaeaceae	*N. nucifera*
					Sword-leaf dogbane (Folium Apocyni)	*Nelumbo nucifera* Gaertn	Apocynaceae	*A. venetum*
				Meigui	Beach rose	*Apocynum venetum*, L.	Rosaceae	*R. rugosa*
				Dahuang	Radix et Rhizoma Rhei; Chinese rhubarb, Rheum	*Rosa rugose*, Thunb.	Polygonaceae	*R. palmatum*, R.tanguticum, and R. officinale
					Glauber’s salt or mirabilite /Natrii Sulphas (Na2S04 10H2O); Chinese mineral stone drug	Rheum palmatum L., Rheum tanguticum Maxim. ex Balf., *and* Rheum officinale Baill	mirabilite	
				Hai Zao (HZ)	Thallus Sargassi	*Mirabilitum Depuratum*	Sargassaceae	*S. pallidum*
					Glycyrrhizae radix; Liquorice root	Sargassum pallidum (Turner) C. Agardh	Fabaceae	*G. uralensis*
14	Azushima et al. (2013)	Bofu-tsu-shosan	Glycyrrhizae radix, Schizonepetae spica, Ephedrae herba, Forsythiae fructus), Others: Platycodi radix, Gypsum fibrosum Atractyloids rhizoma, Rhei rhizoma, Scutellariae radix, Gardeniae fructus, paeoniae radix, cnidii rhizoma, Angelicae radix, Menthae herba, Ledebouriellae radix, Zingilberis rhizoma, Kadinium, Natrium sulfuricum		Glycyrrhizae radix; Liquorice root	*Glycyrrhiza uralensis*, Fisch	Fabaceae	*G. uralensis*
				Jing jie	*Schizonepetae spica;* Japanese catnip	*Glycyrrhiza uralensis*, Fisch	Lamiaceae	*S. tenuifolia*
					Ephedrae herba; Joint-pine, jointfir, Mormon-tea or Brigham tea	*Schizonepeta tenuifolia* (Benth.) Briq; *Nepeta tenuifolia* Benth	Ephedraceae	*E. sinica*
				lianqiao	Weeping forsythia ; golden-bell Forsythia fructus (fruit of Forsythia suspense	*Ephedra sinica *Stapf	Oleaceae	*F. suspensa*
					Chinese bellflower root; balloon flower root; *Platycodi* radix (the root of Platycodon	*Forsythia suspense* (Thunb.) Vahl	Campanulaceae	*P. grandiflorum*
				Duan Shi Gao	main component: CaSO_4_	*Platycodon grandifloras* (Jacq) A. DC		
					*Atractyloides* rhizome	Gypsum fibrosum	Asteraceae/Compositae	*A. macrocephala*
				Da Huang	Rhei rhizome; Chinese rhubarb, Rheum	*Atractylodes macrocephala* Koidz.	Polygonaceae	R*. palmatum*
				Baikal	Skullcap or Chinese skull cap	*Rheum palmatum* L.	Lamiaceae	*S. baicalensis*
				Zhizi	Gardenia; Cape Jasmine	*Scutellaria baicalensis*, Georgi	Rubiaceae	*G. jasminoides*
					Paeoniae radix; Peony root; Chinese peony	*Gardenia jasminoides*, J.Ellis	Paeoniaceae	*P. lactiflora*
					dried root stem of Cnidium officinale; cnidii rhizome	*Paeonia lactiflora* Pall	Apiaceae	
				Duhuo	Angelicae radix	*Cnidium officinale* Makino; *Ligusticum officinale* (Makino) Kitag	Apiaceae	*A. pubescens*
				Bo He	Menthae herba		Lamiaceae	*M. Haplocalycis*
				Fang feng	*Radix Ledebouriella*	*Angelica pubescens* Maxim.	Apiaceae	*S. divaricata*
					Ginger (Zingiberis rhizome)	Mentha canadensis L; *Menthae haplocalyx* Briq	Zingiberaceae	*Z. officinale*
15	Li etal. (2015)	Tang-Nai-Kang:	*Fructus Ligustri* Lucidi, *Spica Prunellae vulgaris, Saururus chinensis*, *Psidium guajava* and Radix ginseng (25:10:15:10	Nuzhenzi	broad-leaf privet; Fructus Ligustri Lucidi	*Saposhnikovia divaricata* (Turcz.) Schischk; *Ledebouriella divaricata* (Turcz.) Hiroe	Oleaceae	*L. lucidum*
					Common self-heal, heal-all, (Spica Prunellae vulgaris)	*Zingiber officinale* Roscoe	Lamiaceae	*P. vulgaris*
					Asian lizard's tail (Saururus chinensis)	*Ligustrum lucidum*i, W.T.Aiton	Saururaceae	*S. chinensis*
					common guava	*Prunella vulgaris* L.	Myrtaceae	*P. guajava*
					Radix ginseng	*Saururus chinensis*, (Lour.) Baill	Araliaceae	*P. ginseng*
16	Chen et al. (2017)	Wendan decoction (WDD)	*Radix Glycyrrhizae* (3 g), *Pericarpium Citri Reticulatae* (9 g), *Poria Cocos* (4.5 g), *Citrus aurantium* (6 g), *Pinellia ternata* (6 g) ad *Caulis bambusae* (6 g)		Glycyrrhizae radix; Liquorice root	*Psidium guajava* L.	Fabaceae	*G. uralensis*
					Pericarpium of mandarin orange (dried and ripe peel)	*Panax ginseng* C. A. Meye	Rutaceae	*C. reticulata*
					Poria cocos, China root	*Glycyrrhiza uralensis*, Fisch	Polyporaceae	*W. extensa*
					Bitter orange,	*Citrus reticulata* *,* *Blanco*	Rutaceae	*C. aurantium*
					crow-dipper	*Wolfiporia cocos* (F.A. Wolf) Ryvarden and Gilb.	Araceae	*P. ternata*
					Caulis Bambusae (Bamboo shavings)	Citrus aurantium L.	Poaceae	*P. nigra*
17	Leong et al. (2013)	Herbal formula MCC:	*Momordica charantia*, the pericarpium of *Citri reticulate* and L-carnitine		Bittermelon; Balsam Pear	*Pinellia ternata*, (Thunb.) Makino	Cucurbitaceae	*M. charantia*
					Pericarpium of mandarin orange (dried and ripe peel)	*Phyllostachys nigravar. henonis* (Mitford) Rendle	Rutaceae	*C. reticulata*
					L-carnitine	*Momordica charantia* L.		
18	Tan et al. (2011)	Chinese herbal extract (SK0506)	*Gynostemma pentaphyllum, Coptis chinensis and Salvia miltiorrhiza* (gypenosides, berberine and tanshinone)	jiaogulan	five-leaf ginseng	*Citrus reticulata* *,* *Blanco*	Cucurbitaceae	*G. pentaphyllum*
				Huang Lian	Chinese goldthread or canker root		Ranunculaceae	*C. chinensis; C. deltoidea* and *C. teeta*
				Danshen	Red sage, Chinese sage	*Gynostemma pentaphyllum* *(Thunb.) Makino*	Lamiaceae	*S. miltiorrhiza*
19	Liu and Shi (2015)	Yi Tang Kang	sugar, *Poria cocos*, atractylodes, *Radix astragali*, red ginseng and other drugs		Poria cocos, China root	*Coptis chinensis* Franch; *Coptis deltoidea* C.Y. Cheng et Hsiao, and *Coptis teeta* Wall	Polyporaceae	*W. extensa*
				Baizhu	obtained from roots of sunflower family	*Salvia miltiorrhiza*, Bunge	Asteraceae/Compositae	*A. macrocephala*
					Red ginseng (produced by steaming and drying fresh and raw ginseng.	*Wolfiporia cocos* (F.A. Wolf) Ryvarden and Gilb	Araliaceae	*P. ginseng*
20	Lim et al. (2019)	SCH	Pharbitish semen; Trogopterorumh Faeces, Cyperih Rhizoma = 2:1:1		Pharbitish Semen (*Pharbitis nill* Seed) picotee morning glory	*Atractylodes macrocephala* Koidz.	Convolvulaceae	*I. nil*
					Trogopterorum Faeces;complex-toothed flying squirrel	Panax ginseng C.A. Meyer	Sciuridae	*T. xanthipes*
					coco-grass, Java grass (Cyperi Rhizoma)	*Ipomoea nil*, (L.) Roth; *Pharbitis nil* (L.) Choisy	Cyperaceae	*C. rotundus*
21	Ahmed et al. (2009)	Marjoram and chicory	Marjoram dry leaves (*Origanum majorana*) and chicory dry leaves (*Cichorium intybus*) (1:5 w/v in water)		Marjoram dry leaves	*Trogopterus xanthipes*, (Milne-Edwards)	Lamiaceae	*O. majorana*
					chicory dry leaves; Common chicory	*Cyperus rotundus* L.	Asteraceae	*C. intybus*
22	Jang et al. (2018)	Gambihwan (GBH1)	Ephedrae Herba; Coicis semen; Menthae herba Gypsum; Alismatis Rhizoma; Crataegi fructus; Arecae semen; Hordei fructus germinatus. GBH2: Ephedrae herba; Coicis semen; Typhae pollen; Castaneae semen; Sinomeni Caulis et Rhizoma; Scutellariae radix		Ephedrae Herba	*Origanum majorana* L	Ephedraceae	*E.sinica*
					Job’s tears seed or adlay; Coix seed; Coicis semen;	*Cichorium intybus* L	Poaceae/Gramineae	*C. lacryma-jobi*
				Bo He	Menthae herba	*Ephedra sinica* Stapf	Lamiaceae	*M. Haplocalycis*
					Alisma; Asian water-plantain; mad-dog weed	*Coix lacryma-jobi* L.	Alismataceae	*A. orientale*
					Single-seeded hawthorn;Hawthorn Berry	Mentha canadensis L; *Menthae haplocalyx* Briq	Rosaceae	*C. monogyna*
					Arecae semen; areca; betel nut, areca nut	*Alisma orientale* (Sam.) Juzep; *Alisma plantago-aquatica*subsp. *orientale* (Sam.) Sam	Palmaceae	*A. catechu*
					Malt Barley Sprout; germinated barley; (Hordei fructus germinates)	Crataegus monogynaJacq	Poaceae/Gramineae	*H. vulgare*
				Sheng Pu Huang	Typha Pollen, Cattail Pollen, Bulrush	*Areca catechu* L	Typhaceae	*T. angustifolia*
					Castaneae semenDried Chestnut	*Hordeum vulgare* L	Sapindaceae	*A. hippocastanum*
				Boi	Sinomeni Caulis et Rhizoma	*Typha angustifolia* L	Menispermaceae	*S. acutum*
				Baikal	Skullcap or Chinese skullcap (Radix scutellariae)	*Aesculus hippocastanum* L	Lamiaceae	*S. baicalensis*
23	Wat et al. (2018)		combination of sylimarin, *Schisandrae Fructus*, Crataegus *Fructus* and *Momordica charantia* (1:1:1:1)		Sylimarin (flavonolignans extracted from the milk thistle *Silybum marianum* (L.)	*Sinomenium acutum* (Thunb.) Rehder and E.H.Wilson	Asteraceae	*S. marianum*
					Magnolia-Vine, Chinese magnolia-vine, schisandra (Schisandrae Fructus)	*Scutellaria baicalensis*, Georgi	Schisandraceae	*S. chinensis*
					Single-seeded hawthorn;Hawthorn Berry	*Silybum marianum*, (L.) Gaertn	Rosaceae	*C. monogyna*,
					Bittermelon; Balsam Pear	*Schisandra chinensis* (Turcz.) Baill	Cucurbitaceae	*M. charantia*
24	Park et al. (2009)	Herbal Complex (HC) extract	Dioscorea Rhizoma, Glycine soja Sieb. et Zucc, Bombycis corpus, Fermented Glycine soja	SanYak	Dioscorea Rhizoma; Chinese Yam	Crataegus monogyna Jacq	Dioscoreaceae	*D. polystachya*
					wild soybean	*Momordica charantia* L.	Leguminosae/Fabaceae	*G. soja*
					Bombycis corpus a drug consisting of the dried larva of silkworm, dead and stiffened due to the infection of fungus *Beauveria bassiana* (Bals.) Vuill	*Dioscorea polystachya*, Turcz.	Cordycipitaceae	*B. bassiana*
					Fermented Glycine soja; cultivated soybean	Glycine max *subsp.* soja (Siebold and Zucc)	Leguminosae/Fabaceae	*G. soja*
						Bombyx Batryticatus (silkworm infected of fungus *Beauveria bassiana* (Bals.) Vuill)		
						*Glycine max* [L.] Merr		

**TABLE 2:** Taxonomic classification of all the polyherbal combinations used in clinical studies against metabolic syndrome.

S. No	References	Name of the herb	Components	Chinese Name	Common name/source	Scientific name	Family	Specie
1	Tian-zhan et al. (2019)	Yiqi Huazhuo Gushen herbal formula	Huang qi (*Astragalus membranaceus*); Huanglian (*Coptis chinensis*), Shengpuhuang (Pollen typhae), Ze Xie (the rhizome of oriental water plantain), Lu Dou Yi (Mung bean peel), Liu Yue Xue (*Serissa serissoides*), Zhi-fuzi (Radix Aconiti lateralis praeparata)	Huang Qi	Mongolian milkvetch; root of Astragalus; Radix astragali	*Astragalus membranaceus* (Fisch.) Bunge; *Astragalus propinquus* Schischkin	Fabaceae	*A. membranace*
				Huang Lian	Chinese goldthread or canker root	*Coptis chinensis* Franch; *Coptis deltoidea* C.Y. Cheng et Hsiao, and *Coptis teeta* Wall	Ranunculaceae	*C. chinensis; C. deltoidea* and *C. teeta*
				Sheng Pu Huang	Typha Pollen	*Typha angustifolia* L	Typhaceae	*T. Angustifolia*
					Cattail Pollen			
					Bulrush			
				Ze Xie	the rhizome of oriental water plantain; Alisma; Asian water-plantain; mad-dog weed	*Alisma orientale* (Sam.) Juzep; *Alisma plantago*-*aquatica* subsp. *orientale* (Sam.) Sam	Alismataceae	*A. orientale*
				Lu Dou Yi ; Hei Dou	Mung bean peel; Soybean meal	*Vigna radiata* (L.) R. Wilczek; *Testa glycinis*	Fabaceae	*V. radiata*, *T. glycinis*
				Liu Yue Xue	Chinese Snow of June Herb;	*Serissa serissoides* (DC.) Druce	Rubiaceae	*S. serissoides*
				Zhi-fuzi	Radix Aconiti lateralis Preparata (Prepared Aconite; Prepared Sichuan Aconite Root; monkshood root)	*Aconitum carmichaelii* Debeaux	Ranunculaceae	*A. carmichaelii*
2	Wang et al. (2013)	Yiqi Huaju Qingli	Huangqi (Radix	Details similar as previous except slight difference in methods of collection of the extracts				
			Astragali)					
			Huanglian (Rhizoma Coptidis)					
			Pu huang (Pollen Typhae)					
			Ze Xie (Artemisiae Rhizoma Alismatis)					
			Lu Dou Yi (Testa Vignae Radiatae), Liu Yue Xue (Serissa Japonica)					
			Fuzi (Radix Aconiti Lateralis Preparata)					
3	Farajbakhsh et al. (2019)	Sesame oil and vitamin E			Sesame oil	*Sesamum indicum* L	Pedaliaceae	*S. indicum*
					Vitamin E	α- tocopherol		
4	Amin et al. (2015b)	Curcuma longa and Nigella sativa	Curcuma longa and Nigella sativa		Turmeric	*Curcuma longa* L	Zingiberaceae	*C. longa*
					Kalonji/black seeds	*Nigella sativa* L	Ranunculaceae	*N. sativa*
5	Yadav et al. (2014)	Diabegon	*Momordica charantia, Gymnema sylvestre*, *Trigonella foenumgraecum*, *Plumbago zeylanica*, *Eugena jambolana, Aegle marmelos, Terminalia chebula, Terminelia balerica, Emblica officinalis, Curcuma longa, Pterocarpus marsupium, Berberis aristata, Cytrullus culocynthis, Cyperus rotondus*, Piper longum, root of Piper longum, Zingiber officinale, and Asphaltum punjabinum		Bittermelon; Balsam Pear	*Momordica charantia* L	Cucurbitaceae	*M. charantia*
					Chirata; Chiretta	*Swertia chirayita* (Roxb.) Buch.-Ham. ex C.B.Clarke	Gentianaceae	*S. chirayita*
					Gurmar	*Gymnema sylvestre* (Retz.) Schult	Apocynaceae	*G*. sylvestre
					Fenugreek	*Trigonella foenum-graecum* L	Fabaceae/Leguminosae	*T. foenum-graecum*
					Plumbago; Ceylon leadwort, doctorbush or wild leadwort	*Plumbago zeylanica* L	Plumbaginaceae	*P. zeylanica*
					Jamon; Java Plum	*Eugenia jambolana* Lam;	Myrtaceae	*S. cumini*
						*Syzygium cumini* (L.) Skeels		
					Bael, Bengal Quince	*Aegle marmelos* (L.) Correa	Rutaceae	*A. marmelos*
					Chebulic myrobalan, haritali; black- or chebulic myrobalan	*Terminalia chebula* Retz	Combretaceae	*T. chebula*
					Belleric; bahera or beleric or bastard myrobalan	*Terminalia bellirica* (Gaertn.) Roxb	Combretaceae	*T. bellirica*
					Emblic myrobalan	*Phyllanthus emblica*	Phyllanthaceae	*P. emblica*
						L.; *Emblica officinalis*		
					Turmeric	*Curcuma longa* L	Zingiberaceae	*C. longa*
					Malabar kino	*Pterocarpus marsupium *Roxb	Fabaceae	*P. marsupium*
					Indian Barberry, Tree Turmeric	*Berberis aristate* DC.	Berberidaceae	*B. aristata*
					Colocynth, Bitter apple, wild gourd	*Citrullus colocynthis*	Cucurbitaceae	*C. colocynthis*
						(L.) Schrad		
					Coco-grass, Java grass, nut grass, purple nut sedge	*Cyperus rotundus*	Cyperaceae	*C. rotundus*
						L		
					Long pepper; Indian long pepper or pipli	*Piper longum*	Piperaceae,	*P. longum*
						L		
					Pippalimula (*root* of *Piper longum*)	*Piper longum*<	Piperaceae,	*P. longum*
						L		
					Ginger	*Zingiber officinale*	Zingiberaceae	*Z. officinale*
					Asphaltum punjabinum; Shilajatu; Shilajit, Mineral Pitch, Asphlat (Some researchers hypothesize that shilajit is produced by the decomposition or humification of latex and resin-bearing plant material from species such as *Euphorbia royleana* and *Trifolium repens* over a period of centuries)	—	blackish-brown powder or an exudate from high mountain rocks	
6	Yang et al. (2014b)	Modified, Lingguizhugan decoction (MLD)+ weekend fasting	Dangshen (Radix Codonopsis) 20 g, Guizhi (Ramulus Cinnamomi) 12 g, Fuling (Poria) 30 g, Baizhu (Rhizoma Atractylodis Macrocephalae) 15 g, Gancao (Radix Glycyrrhizae) 6 g; Dahuang (Radix Et Rhizoma Rhei Palmati) 9 g	Dangshen	Radix *Codonopsis pilosulae* (mixture)	*Codonopsis pilosula* (Franch.) Nannf	Campanulaceae	*C. pilosula, C. pilosula var. modesta and C. tangshen*
				GuiZhi	Ramulus Cinnamomi (obtained from dried twigs of *Cinnamomum cassia* (L.) Presl,	*Cinnamomum cassia* (L.) Presl	Lauraceae	C. cassia
				Fu Ling	Poria, Hoelen, Indian bread, Poria, Tuckahoe	*Wolfiporia cocos* (F.A. Wolf) Ryvarden & Gilb	Polyporaceae	*W. extensa*
				Gan Cao	Liquorice root; Radix Glycyrrhizae	*Glycyrrhiza uralensis,* Fisch	Fabaceae	*G. uralensis*
				Baizhu, Atractylodes	obtained from roots of *Atractylodes Macrocephala* Koidz	*Atractylodes macrocephala* Koidz	Asteraceae	
				Dahuang	Radix et Rhizoma Rhei; Chinese rhubarb, Rheum	Rheum palmatum L., Rheum tanguticum Maxim. ex Balf., and Rheum officinale Baill	Polygonaceae	*R. palmatum,* R.tanguticum and R. officinale
7	Yu et al. (2018)	Dahuang Huanglian Xiexin Decoction (JTTZ)	Aloe vera, *Coptis chinensis*, Rhizoma Anemarrhenae, red yeast rice, Momordica charantia, Salvia miltiorrhiza, Schisandra chinensis, and dried ginger	Luhui	Aloe vera	*Aloe vera*, (L.) Burm.f	Asphodelaceae	*A. vera*
				Huanglian	Chinese goldthread	*Coptis chinensis*, Franch	Ranunculaceae	*C. chinensis*
				Zhi mu	Rhizoma Anemarrhena	*Anemarrhena asphodeloides*, Bunge	Asparagaceae	*A. asphodeloides*
				Hong qu	red yeast rice (purple fermented rice, cultivated with the mold *Monascus purpureus*)	*Monascus purpureus*, (Went, 1895)	Monascaceae	*M. purpureus*
				Kugua	Bittermelon; Balsam Pear	*Momordica charantia* L	Cucurbitaceae	*M. charantia*
				Danshen	Red sage, Chinese sage	*Salvia miltiorrhiza*, Bunge	Lamiaceae	*S. miltiorrhiza*
				Wuweizi	Magnolia-vine, Chinese magnolia-vine, schisandra	*Schisandra chinensis* (Turcz.) Baill	Schisandraceae	*S. chinensis*
				Ganjiang	Dried ginger	*Zingiber officinale* Roscoe	Zingiberaceae	*Z. officinale*
8	Rozza et al. (2009)	Armolipid Prev, Rottapharm, Monza, Italy) + dietary intervention	Combination of *Ortosiphon staminensis*, with policosanol (dietary supplement), red yeast rice extract, berberine, folic acid and coenzyme Q10		*Misai, kucing* and *kumis kucing*	*Orthosiphon stamineus* Benth	Lamiaceae	*O. stamineus*
					policosanol (mixture of alcohols isolated from Cuban sugar cane wax	*Saccharum officinarum*	Poaceae	*S. officinarum*
						L		
					Red yeast rice extract (purple fermented rice, cultivated with the mold *Monascus purpureus*)	*Monascus purpureus*	Monascaceae	*M. purpureus*
						(Went, 1895)		
					Berberine (chemical in Berberis genus)	chemical		
					Folic acid (obtained from food source)	chemical		
					coenzyme Q10	chemical	coenzyme	
9	Castellino et al. (2019)	Cynara cardunculus (L.) subsp. scolymus Hayek-based nutraceutical, named Altilix	*Cynara cardunculus* (L.) subsp. scolymus Hayek; Chlorogenic Acid and Luteolin		Artichoke; cardoon	*Cynara cardunculus* (L.)	Asteraceae	C. cardunculus (scolymus Hayek)
					Chlorogenic Acid (ester of caffeic acid and-quinic acid)	compound: C16H18O9	dietary polyphenol	
					Luteolin	Chemical compound: C15H10O6	flavone, a type of flavonoid,	
10	Panahi et al. (2015)	curcuminoids (Curcumin C3 Complex®, Sami Labs LTD, Bangalore, India); piperine (Bioperine®; Sami Labs LTD, Bangalore, India) was added to enhance Bioavailability	(95% curcuminoids (70% is curcumin; remaining demethoxycurcumin and bisdemethoxycurcumin in patented ratio. Curcuminoids obtained from turmeric 5% piperine (obtained from black pepper		Curcuminoids (curcumin; demethoxycurcumin and bisdemethoxycurcumin)	*Curcuma longa* L	Zingiberaceae	*C. longa*
					Piperine	*Piper nigrum* L	Piperaceae	*P. nigrum*
11	Panahi et al. (2014)	Curcuminoids (piperine was added to enhance Bioavailability) (95% curcuminoids, of which at least 70% is curcumin)	(95% curcuminoids (70% is curcumin; remaining demethoxycurcumin and bisdemethoxycurcumin in patented ratio. Curcuminoids obtained from turmeric 5% piperine (obtained from black pepper		curcuminoids (curcumin; demethoxycurcumin and bisdemethoxycurcumin)	*Curcuma longa* L	Zingiberaceae	*C. longa*
					Piperine	*Piper nigrum* L	Piperaceae	*P. nigrum*
12	Verhoeven et al. (2015)	Red yeast rice (obtained by culturing the yeast *Monascus purpureus* on rice) and olive extract	Red yeast rice (obtained by culturing the yeast *Monascus purpureus* on rice) and olive extract		red yeast rice (Purple fermented rice, cultivated with the mold *Monascus purpureus*)	*Monascus purpureus*, (Went, 1895)	Monascaceae	*M. purpureus*
					olive extract	*Olea europaea* L	Oleaceae	O. europaea
13	He et al. (2007)	Yiqi Sanju Formula	Details not available as paper is in Chinese					
14	Lee et al. (2012)	Red yeast rice, bitter gourd, chlorella, soy protein, and licorice	Red yeast rice, bitter gourd, chlorella, soy protein, and licorice		Red yeast rice	*Monascus purpureus*, (Went, 1895)	Monascaceae	*M. purpureus*
					Bitter gourd	*Momordica charantia* L	Cucurbitaceae	*M. charantia*
					Green algae	Chlorella	Chlorellaceae	
					Soy protein (isolated from soybean)	*Glycine* max (L.) Merr	Fabaceae	*G. max*
					Licorice	*Glycyrrhiza glabra* L	Fabaceae/Leguminosae	*G. glabra*
15	Nagata et al. (2012)	Keishibukuryogan (Guizhi-Fuling-Wan)	Cinnamomi Cortex, Paeoniae Radix, Moutan Cortex, Persicae Semen, and Hoelen	Guizhi	Cinnamomi cortex (dried bark of *Cinnamomum verum*); Chinese cinnamon	*Cinnamomum verum* J.Presl	Lauraceae	*C. veruum*
				Shaoyao	Paeoniae Radix; Peony root; Chinese peony	Paeonia lactiflora Pall	Paeoniaceae	*P. lactiflora*
				Mudanpi	Moutan Cortex	*Paeonia* x *suffruticosa* Andrews	Paeoniaceae	*P. × suffruticosa*
				Taoren	Persicae Semen; fruit kernel of Peach	*Prunus persica* (L) Batsch	Rosaceae	*P. persica*
				Fuling	Hoelen (dried sclerotia of *Wolfiporia cocos;*	*Wolfiporia cocos* (F.A. Wolf) Ryvarden & Gilb	Polyporaceae	*W. extensa*

**TABLE 3:** Summary of metanalysis of Poly herbal combinations used in animal-based models of Metabolic syndrome.

S. No	Polyherbal combination	Model/animal/treatment duration	Parameters assessed: 5 = glucose/FBG, TG, HDL-C, BP and central obesity (weight, BMI, HC and WC). Parameters met: BMI [WC, HC], BP, HDL, TG, FBG. Additional: TC, LDL	Other parameters related to MetS	Score of study MetS parameters assessed >3 = 1; ≤3 = 0	Score for effects (3/5: Good) = score 1; <3/5 (not so good) = score 0	Concentration given	Quality control	Chemical classification	References
1	*Curcuma longa*, *Salacia reticulate*, *Gymnema sylvestre*, *Emblica officinalis*, *Terminalia chebula*	High fructose diet/Wistar rats/3 weeks	Assessed: 5/5 = Body weight, abdominal waist, BP, glucose, TG, HDL-C, TC, LDL and VLDL. Met: 5/5 = Lowered Body weight, abdominal waist and BMI, reduced BP, AI, improved FBG and OGTT, reduced TG, increased HDL-C. Also, TC, LDL and VLDL reduced	Reduced SGOT, SGPT, Uric acid, MDA. Reduced gastrocnemius muscle weight and fat pads. Reduced infiltration of inflammatory cells and fat accumulation in liver and pancreas	1	1	Yes	No (Purchased from registered company (References no: SR/KN/CL/1/2003)	No	Thota et al. (2014)
2	DHSGT: *Glycyrrhizae uralensis* Fischer (40 g), *Rheum undulatum* Linne (80 g), *Prunus persica* Linne (60 g), *Cinnamomum cassia* Presl (40 g), and Natrii Sulfas (40 g)	HFD-induced obesity/C57BL/6 J mice/7 weeks	Assessed: 5/5 = Body weight, BP, TG, HDL, Glucose. TC and LDL, Met: 5/5 = Reduced body weight (Reduced liver weight and adipose tissue mass, adipocyte size), BP, TG, glucose and increased HDL-c. TC and LDL-c reduced	Decreased serum leptin and leptin mRNA expression. increased mRNA expression of peroxisome proliferator activated receptor-gamma, uncoupling protein-2, and adiponectin in visceral adipose tissue of HFD mice. Inhibition of porcine pancreatic lipase and ACE activities *in vitro*	1	1	Yes	No	No	Sung et al. (2014)
3	Huang-lian-jie-du-tang: Rhizoma coptidis, Radix scutellariae, Cortex phellodendri and Fructus gardeniae (3:2:2:3)	Obese-diet (2% fat, 10% sucrose, 6% salt and 8% defatted milk powder) and drinking water (20% sucrose solution) ad libitum/Wistar male rats/12 weeks	Assessed: 5/5 = BP, body weight, FBG, fasting insulin, and insulin resistance index, TG, HDL-C, LDL-c. Met: 5/5 **=** Reduction in body weight, BP, FBG, fasting insulin and insulin resistance index, TG levels reduced, and HDL-c increased. LDL-C reduced	inhibited the activation of NF-kB and reduced serine phosphorylation of IRS-1	1	1	Yes	Yes	No	Li et al. (2013)
4	RGPM: Red ginseng and *Polygoni Multiflori* Radix (1:1)	High fructose/SD rats/6 weeks	Assessed: 5/5 = body weight, Glucose, BP, TG, HDL-c. TC and LDL-c. Met: 5/5 = Reduced body weight and epididymal fat pads weight, reduced TG, systolic BP and increased HDL-c, OGTT improved. TC and LDL-c reduced	reduced leptin, CRP and glutamic-oxaloacetic transaminase, Decreased VCAM-1, ICAM-1, E selectin, MCP-1 and improved PPAR-γ expression. lipid droplets in liver decreased	1	1	Yes	No (Commercially available product was used)	No	Kho et al. (2016)
5	Modified lingguizhugan decoction with dietary restriction and exercise. [*Poria cocos* Wolf, *Cinnamomumcassia* Presl, *Atractylodes lancea* DC., *Glycyrrhiza uralensis* Fisch., *Codonopsis pilosula*, Nannf. and *Rheum palmatum* L] (ratio of 12:9:6:6:9:9)	HFD for 12 weeks (30% HFD + dietary restriction ± 45 min swim)/adult SD male rats/1 week after HFD for subsequent 12 weeks	Assessed: 5/5 = body weight, TG, HDL, BP, blood glucose. Met: 5/5 = reduced body weight, TG, BP, blood glucose and insulin levels, Increased HDL. Reduced TC, LDL, adipose and liver tissue weight	Reduced serum FFA, AST, ALT and ALP and TNF-α, leptin in serum and liver	1	1	Yes	Yes	Yes	Yao et al. (2017b)
6	*Curcuma longa* and *Nigella sativa*	Fructose fed rats (60% fructose in diet + white flour instead of wheat flour) for 6 weeks/SD rats/6 weeks	Assessed: 5/5 = body weight, BP, Fasting serum insulin, FBG, HDL, and TG, Met: 4/5 = Reduced BP, TG, FBG, increased HDL. Reduced LDL, TC and insulin	CRP reduced	1	1	Yes	No	Yes	Amin et al. (2015a)
7	Soybean meal and probiotics (*Bifidobacterium, longum* (BB536)	Obese Zucker rats/Rat/100 days (14.2 weeks)	Assessed: 4/5 = Body weight, TC, TG, HDL and glucose. Met: 4/5 = Reduced weight gain (reduced liver weight and fat), FBG and insulin, TG and Increased HDL. Reduced TC and LDL.	Reduced food intake, ALT, GGT, ALP	1	1	Yes	No but the diet was purchased commercially	No	Mounts et al. (2015)
8	ACE: *Artemisia iwayomogi* and *Curcuma longa* (1:1)	HFD (10 weeks)/C57BL/6/male mice/10 weeks	Assessed: 4/5 = Body weight, TG, FBG, HDL-C (TC and LDL-c). Met: 4/5 = Reduced body weight (reduced liver weight, epididymal, retroperitoneal, and visceral adipose tissues. Reduced adipocyte size, TC and TG in liver), reduced serum TG, FBG and increased HDL. Reduced LDL-c and TC	PPAR-γ, fatty acid synthase; SREBP-1c; and PPAR-α	1	1	Yes	Yes	Yes	Lee et al. (2015b)
9	Fu Fang Zhen Zhu Tiao Zhi formula (FTZ): *Ligustrum lucidum* W.T. Aiton, fructus; *Atractylodes macrocephala* Koidz., rhizoma; *Salvia miltiorhiza* Bunge, radix; *Coptis chinensis* Franch., rhizoma; *Panax notoginseng* F.H.Chen, radix; *Eucom- mia ulmoides* Oliv., cortex; *Cirsium japonicum* Fisch. ex DC., radix; *Citrus medica* var. *sarcodactylus* Swingle, fructus	HFD and insulin resistant HepG2 cell lines/Male SD rats/8 weeks	Assessed: 4/5 = Body weight, FBG, TG, HDL-c, TC. Parameters met: 4/5 = Reduced body weight, FBG (HOMA-IR index), TG increased HDL-c. reduced TC.	Increased PI3K p85 mRNA expression in the adipose tissues. Reduced glucose content, PI3K p85 mRNA and IRS1 protein expression upregulated in insulin resistant HepG2 cells	1	1	Yes	Yes	Yes	Hu et al. (2014)
10	Erchen decoction: Pericarpium Citri Reticulatae (9 g), Rhizoma Pinelliae (9 g), Poria (6 g) and Radix Glycyrrhizae (3 g)	HFD for 10 weeks/Male C57BL/6J mice/4 weeks	Assessed: 4/5 = glucose, TG, HDL, obesity, Met: 3/5 = reduced Body weight, Abdominal circumference, FBG and improved OGTT, no effect on insulin levels. Reduced TG but no effect on HDL-c and LDL-c. Reduced TC.	Increased CDKAL1 expression in the liver, visceral and subcutaneous adipose tissues increased, improved islet cell function to secrete more insulin	1	0	Yes	No	No	Gao et al. (2015)
11	CPQ: Curcumin, Piperine and Quercetin in a ratio (94 :1:5)	HFD and Low-Dose Streptozotocin (8 weeks)/Albino female Wistar rats/28 days	Assessed: 4/5 = body weight, Glucose, TG, HDL (LDL and TC also assessed), Met: 3/5, improved glucose tolerance, reduced TG and increased HDL. LDL-c and TC reduced	Increased catalase, glutathione, and SOD. Decreased granular degeneration in diabetic liver	1	1	yes	yes	Yes	Gao et al. (2015)
12	Extracts of *Salvia miltiorrhiza*+ *Gardenia jasminoides*	HFD/SD male rats/4 weeks	Assessed: 4/5 **=** Body weight, Serum glucose levels, TG, HDL-c (TC, and LDL-C). Met: 3/5 = Reduced serum TC, TG, body weight (reduced visceral fat mass), glucose, enhanced insulin sensitivity. TC and LDL-c reduced	Reduced Serum non-esterified fatty acids, ALT and AST, adipokines, TNF-α and IL-6. Increased leptin in adipose tissue. Enhanced leptin expression	1	0	Yes	Yes	Yes	Tan et al. (2013)
13	SUB885C: Fructus Crataegi, Folium Nelumbinis, Folium Apocyni, Flos *Rosa rugosae*, Radix et Rhizoma Rhei, Depuratum mirabilitum, Thallus Sargassi, and honey fried Radix Glycyrrhizae	ApoE*3Leiden.CETP transgenic mice with mild hypercholesterolemia on semi-synthetic modified Western-type diet (0.2% cholesterol, 15% saturated fat and 40% sucrose; Cell line: 3T3-L1 preadipocyte/Mice/4 weeks	Assessed: 3/5 = Body Weight, TG, HDL-c. also TC, Met: 2/5 = Reduced TG, increased HDL-c. Also reduced TC	Reduced CETP, vLDL-c and TGs. Stimulated lipolysis and inhibited adipogenesis in 3T3-L1 cells	0	0	Yes	Yes	No	Wei et al. (2012)
14	Bofu-tsu-shosan formula: *Glycyrrhizae* radix, *Schizonepetae spica*, Ephedrae herba, Forsythiae fructus) Others: Platycodi radix, Gypsum fibrosum Atractyloids rhizoma, Rhei rhizoma, Scutellariae radix, Gardeniae fructus, paeoniae radix, cnidii rhizoma, Angelicae radix, Menthae herba, Ledebouriellae radix, Zingilberis rhizoma, Kadinium, Natrium sulfuricum	KKAy mice 9 weeks of age/mice/8 weeks 4.7% BOF (Chronic model), 14 weeks KKAy mice/male mice/5,000 mg/kg BOF dissolved in 1ml of distilled water per 100 g of body weight for 1 day (Acute model)	Assessed: 4/5 = obesity with marked visceral fat, blood glucose, HDL and BP, Met: 2/5 = Lowered Body weight, obesity, BP. LDL reduced. No effect on non-FBG, TC, HDL.	Food intake reduced; White adipose tissue (weight and cell size decreased); expression of genes increased: adiponectin and PPAR receptors; reduction in plasma acylated-ghrelin genes expression (antihypertensive effect)	**1**	0	Yes	No, but ingredients were recruited from commercial manufacturers	No	Azushima et al. (2013)
15	Tang-Nai-Kang: Fructus *Ligustri Lucidi*, *Spica Prunellae* vulgaris, *Saururus chinensis*, *Psidium guajava* and Radix ginseng (25:10:15:10	SHR. Cg-Lepr^cp^/ND_mcr_ (SHR/cp) for disease and WKY rats for control/male rat 7 weeks/)/low and high dose 2 weeks	Assessed: 4/5 = BP, sugar, SBP, bodyweight and fat, TG, Met: 4/5 = reduced SBP, body weight and fat mass, FBG, insulin levels. Insulin resistance (OGTT and ITT) was reduced. TC levels did not reduce significantly	AST, ALT, FFA reduced. Gene expression of NAD+ -dependent deacetylase E10 and genes related to fatty acid oxidation were markedly up- regulated in the muscle, liver and adipose tissues	1	1	Yes	No but the process was carried out by Sichuan Medco Pharmaceutical Limited Corporation (Deyang, China), hence some validation is expected	Yes	Li et al. (2015)
16	Wendan decoction: Radix Glycyrrhizae (3g), Pericarpium Citri Reticulatae (9g), Poria Cocos (4.5g), Citrus Aurantium (6g), *Pinellia*, *ternata* (6g ) and Caulis Bambusae (6g)	High-sugar-fat-diet (15 weeks) and high-fat emulsion (2 weeks)/Wistar male rat/2 weeks	Assessed:3/5 = abdominal perimeters, serum insulin HOMA-IR, HDL. Met: 3/5 = decrease in abdominal perimeters and serum insulin levels, increases in HDL levels, Recovered the HOMA-IR to the control level	pathway analysis and molecular docking simulation	0	1	Yes	Yes	Yes	Chen et al. (2017)
17	MCC: *Momordica charantia*, the pericarpium of Citri reticulate and L-carnitine Dosage: 6 g/kg	HFD/female ICR mice/8 weeks	Assessed: 4/5 = weight gain, FPG and glucose intolerance, insulin sensitivity, TG, HDL (LDL also assessed). Met: 2/5 = reduced TG, FPG, glucose intolerance and Insulin sensitivity index, LDL/HDL ratio and TC levels also reduced	Mitochondrial coupling efficiency of skeletal muscle was improved and reduced carnitine palmitoyl CoA transferase activity	1	0	Yes	No, but commercial preparation was manufactured and supplied by Infinitus (China) Company Ltd., Guangzhou, China	No	Leong et al. (2013)
18	SK0506: *Gynostemma pentaphyllum*, *Coptis chinensis* and *Salvia miltiorrhiza* (gypenosides, berberine and tanshinone)	HFD/Male SD rats/4 weeks	Assessed: 3/5 **=** Body weight, FBG, TG, TC. Parameters met: 3/5 = Lowered body weight, visceral fats, TG, slightly reduced FBG. (Reduced insulin level and NAFA, improved impaired glucose tolerance and glucose infusion rate). TC reduced	Enhanced GLUT4 expression in adipose tissue, enhanced insulin mediated glucose uptake in red quadriceps and white gastrocnemius skeletal muscles, enhanced glycogen synthesis	0	1	No (but yield is given. It seems all powders were taken in equal ratio)	Yes	Yes	Tan et al. (2011)
19	Yi Tang Kang: sugar, *poria cocos, Atractylodes*, radix *Astragali*, red ginseng and other drugs	MS spleen deficiency syndrome rats with HFD and low dose intraperitoneal injection of streptozocin/Male Wistar rats/10 weeks	Assessed: 4/5 = weight gain, FBG, TG, HDL-c. Met: 3/5 = Reduced FBG and TG and increased HDL-c. Reduced insulin levels, insulin resistance (IR) and lSI	Upregulation of Carboxylesterase and retinal guanylate cyclase 2 precursors. Downregulation of IgG, carnitine acetyltransferase, tubulin beta 5, and Gan Lu sugar binding protein C. protein tyrosine kinase, beta glucosidase	1	1	No	No	No	Liu and Shi, (2015)
20	SCH: Pharbitish semen, Trogopterorumh faeces, Cyperih Rhizoma (2:1:1)	HFD mouse model, 3T3-L1and HepG2 cells/Male C57BL/6J/mice/15 weeks	Assessed: 3/5 = Glucose and insulin, TG and TC levels. Parameters met: 3/5 = Reduced glucose levels and insulin levels (HOMA-IR index reduced), Reduced TC and TG.	Regulated adipogenic gene expression, proteins involved in energy metabolism (in maturated 3T3-L1 cells). Increased phosphorylated AMP activated protein, as well as attenuated insulin resistance and hepatic steatosis, improved glucose facilitation by GLUT2 externalization. in FFA-induced steatotic HepG2 cells	0	1	Yes	No	No	Lim et al. (2019)
21	Marjoram and chicory Marjoram dry leaves (*Origanum majorana*) and chicory dry leaves (*Cichorium intybus*) (1:5 w/v in water)	HFD/female SD albino rats/4 weeks	Parameters assessed: 3/5 = Body weight gain, TG, HDL-c (Additional: TC, LDL-c, VLDL-c, adipose tissue weight). Parameters met: 3/5 **=** lowered weight gain (Adiposity index and FER), reduced TG, and increased HDL-c; Adipose tissue weight, TC, LDL-c, VLDL-c also reduced	decreased ALT and AST. increased serum free T4 and T3 hormones	0	1	Yes	No	No	A. Ahmed et al. (2009)
22	Gambihwan (GBH1): Ephedrae Herba; Coicis semen; Menthae herba Gypsum; Alismatis Rhizoma; Crataegi fructus; Arecae semen; Hordei fructus germinatus GBH2: Ephedrae herba; Coicis semen; Typhae pollen; Castaneae semen; Sinomeni Caulis et Rhizoma; Scutellariae radix	Model: HFD-induced obese mice/C57BL/6 mice (4 weeks old)/8 weeks	Assessed: 4/5 = Body weight Glucose, TG, HDL Met: 2/5 **=** Reduced body weight, FBG, insulin levels, Improved OGTT. No effect on HDL. Decrease in TC, liver and fat weight	serum inflammatory and hepatic enzyme levels diminished. suppressed lipid accumulation	1	0	Yes	No	No	Jang et al. (2018)
23	Sylimarin, *Schisandrae* Fructus, *Crataegus* Fructus and *Momordica charantia* (1:1:1:1)	HFD and Cell lines: 3T3-L1, Caco-2 and HepG2 cell line/C57Bl/6 male mice/8 and 12 weeks	Assessed: 4/5 = body weight (fat pad weight to body weight ratios; liver weight to body weight ratios); TG, glucose, insulin. TC, LDL-c also assessed. Met: 1/5 = reduced diet-induced increase in body weight and fat pad mass, reduced diet-induced increase in liver weight, liver lipid, and plasma lipid. No Effect on glucose and insulin. reduced liver TC and TG. Reduced TC and LDL-c	Improved Plasma adiponectin level, reduced inflammation (reduced mac-3 expression) in liver. Inhibitory effects on 3T3-L1 preadipocytes differentiation inhibited the glucose uptake Inhibited fatty acid uptake prevented the cholesterol uptake	0	0	Yes	Yes	Yes	Wat et al. (2018)
24	Herbal Complex extract: *Dioscorea* Rhizoma, *Glycine soja* Sieb. et Zucc, *Bombycis corpus*, Fermented *Glycine* soja	HFD-low dose STZ-induced diabetes/Rat/-	Assessed: 2/5 = Body weight, food intake and food efficiency ratio, FBG, OGTT. Met: 2/5 = Decrease body weight, improved OGTT and reduced FBG	invitro assay: α-glucosidase inhibition (antidiabetic mechanism); protein tyrosine phosphatase 1β (antidiabetic and antiobesity mechanism)	0	0	Yes	Not known (Korean language)	Yes	Park et al. (2009)

Abbreviations: 24hTP, 24 h total urinary protein; 2hPPG, 2 h post prandial glucose; AST, aspartate aminotransferase; ALT, alanine transaminase; ALP, Alkaline phosphatase; BP, blood pressure; BMI, body mass index; CDKAL, CDK5 Regulatory Subunit Associated Protein 1 Like 1); CETP, cholesteryl ester transfer protein; CRP, C-reactive protein; FBG, fasting blood glucose; FFA, free fatty acid; GLUT-4, glucose transporter 4; GGT, glutamyl-transferase; HC, hip circumference; HDL-C, high density lipoproteins; HFD, high fat diet; HOMA-IR, homeostatic model assessment for insulin resistance; ICAM-1, intercellular adhesion molecules; IRS-1, Insulin receptor substrate 1; KKAy, cross between diabetic KK and lethal yellow; LDL, low density lipoprotein; Monocyte chemoattractant protein-1; MAU/*MA*, microalbuminuria; MDA, malondialdehyde; NAFA, non-esterified fatty acids; NF-kB, Nuclear Factor kappa-light-chain-enhancer of activated B cells; PI3K Phosphoinositide 3-kinase SREBP, sterol regulatory element-binding transcription factor; PPAR-γ/a, Peroxisome proliferator-activated receptor gamma/alpha; SD Rats: Sprague Dawley rats; SGOT: Serum glutamic oxaloacetic transaminase; SGPT: Serum glutamic pyruvic transaminase; SOD, superoxide dismutase; TC, total cholesterol; TG, triglycerides; TNF, Tumor necrosis factor; vLDL, very low density lipoprotein; UACR, urea creatinine albumin ratio; vCAM-I, Vascular cell adhesion molecule 1; WC, waist circumference; WHR, waist hip ratio; WKy, Wistar Kyoto.

**TABLE 4:** Qualitative scoring of studies on polyherbal combinations used in animals of Metabolic Syndrome models.

	References	Dosage of herb provided	Components and rationale for dosing	animal ethical approval, Yes = 1, No = 0	Euthanasia protocol mentioned/followed, Yes = 1, No = 0	Model validated for MetS	Positive control used, Yes = 1, No = 0	Met S parameters assessed >3 = 1; <3 = 0	Effect 3/5 parameters met = good effect (score 1) <3/5 = not so good (score 0)	Total score for Quality, 8	Link
1	Thota et al. (2014)	1	1	1	1	1	0	1	1	7	https://citeseerx.ist.psu.edu/viewdoc/download?doi=10.1.1.637.1093&rep=rep1&type=pdf
2	Sung et al. (2014)	1	1	1	1	1	0	1	1	7	https://www.ncbi.nlm.nih.gov/pmc/articles/PMC4193160/
3	Li et al. (2013)	1	1	1	0	1	1	1	1	7	https://www.ncbi.nlm.nih.gov/pmc/articles/PMC3695866/
4	Kho et al. (2016)	1	1	1	0	1	1	1	1	7	https://www.ncbi.nlm.nih.gov/pmc/articles/PMC4784406/pdf/12906_2016_Article_1063.pdf
5	Yao et al. (2017b)	1	1	1	1	1	0	1	1	7	https://link.springer.com/article/10.1186/s12906-017-1557-y
6	Amin et al. (2015a)	1	1	1	0	1	1	1	1	7	https://journals.lww.com/cardiovascularpharm/Abstract/2015/02000/Coadministration_of_Black_Seeds_and_Turmeric_Shows.12.aspx
7	Mounts et al. (2015)	1	1	1	1	1	0	1	1	7	https://www.researchgate.net/publication/281189904_Feeding_Soy_with_Probiotic_Attenuates_Obesity-Related_Metabolic_Syndrome_Traits_in_Obese_Zucker_Rats
8	Lee et al. (2015b)	1	1	1	0	1	0	1	1	7	https://www.ncbi.nlm.nih.gov/pmc/articles/PMC4609822/
9	Hu et al. (2014)	1	1	1	0	1	1	1	1	7	https://www.ncbi.nlm.nih.gov/pmc/articles/PMC3943467/
10	Gao et al. (2015)	1	1	1	1	1	1	1	0	7	https://www.hindawi.com/journals/ecam/2015/501272/
11	Kaur and C, (2012)	1	1	1	0	1	1	1	1	7	https://www.ncbi.nlm.nih.gov/pmc/articles/PMC3317057/
12	Tan et al. (2013)	1	1	1	1	1	1	1	0	7	https://www.ncbi.nlm.nih.gov/pmc/articles/PMC3588405/pdf/ECAM2013-306738.pdf
13	Wei et al. (2012)	1	1	1	1	1	1	0	0	6	https://journals.plos.org/plosone/article?id=10.1371/journal.pone.0030332
14	Azushima et al. (2013)	**1**	**1**	**1**	**1**	**1**	**0**	**1**	**0**	6	**https://journals.plos.org/plosone/article/comments?id=10.1371/journal.pone.0075560**
15	Li et al. (2015)	1	1	1	0	1	0	1	1	6	https://journals.plos.org/plosone/article?id=10.1371/journal.pone.0122024
16	Chen et al. (2017)	1	1	1	0	1	1	0	1	6	https://pubs.rsc.org/en/content/articlepdf/2017/ra/c7ra09779dSupplementary reference: http://www.rsc.org/suppdata/c7/ra/c7ra09779d/c7ra09779d1.pdf
17	Leong et al. (2013)	1	1	1	1	1	0	1	0	6	https://pdfs.semanticscholar.org/670a/eb206f240938b3299e6a18e2fdd97c43ae70.pdf
18	Tan et al. (2011)	1	1	1	0	1	1	0	1	6	https://www.researchgate.net/publication/47447592_Chinese_herbal_extracts_SK0506_as_a_potential_candidate_for_the_therapy_of_the_metabolic_syndrome
19	Liu and Shi, (2015)	1	0	0	0	1	1	1	1	5	https://pubmed.ncbi.nlm.nih.gov/25902033/
20	Lim et al. (2019)	1	1	1	0	1	0	0	1	5	https://www.nature.com/articles/s41598-019-45099-x
21	A. Ahmed et al. (2009)	1	1	0	1	1	0	0	1	5	http://citeseerx.ist.psu.edu/viewdoc/download?doi=10.1.1.321.1771&rep=rep1&type=pdf
22	Jang et al. (2018)	1	0	1	0	1	0	1	0	4	https://www.hindawi.com/journals/ecam/2018/5614091/
23	Wat et al. (2018)	1	1	1	1	1	0	0	0	4	https://pubmed.ncbi.nlm.nih.gov/29655677/
24	Park et al. (2009)	0	0	0	0	1	1	0	0	2	https://www.researchgate.net/publication/288976056_Effects_of_herbal_complex_on_blood_glucose_in_streptozotocin-induced_diabetic_rats_and_in_mice_model_of_metabolic_syndrome

Out of 26 clinical trial articles, 15 articles matched our main objective, and their meta-analysis is presented in Table 5 according to SPIDER model with references. Supplementary Table S1 is attached to shows the analysis by SPICE protocol along with information about other targets met besides the 5 parameters of MetS. Table 6 summarizes the qualitative scoring based on a checklist as mentioned in analysis section along with the online link available for the same. Out of 15 polyherbal combinations that were reviewed three formulations were able to modify 4 MetS parameters clinically. They include Yiqi Huazhuo Gushen herbal formula (Tian-zhan et al., 2019), Yiqi Huaju Qingli Formula (Wang et al., 2013), Sesame oil and vitamin E (Farajbakhsh et al., 2019). Six polyherbal combinations were able to reduce three out of 5 standard MetS parameters. The combinations included, *Curcuma longa* and *Nigella sativa* (Amin et al., 2015b), Diabegon (Yadav et al., 2014), modified Lingguizhugan decoction (MLD)+ weekend fasting (Yang et al., 2014a), Dahuang Huanglian Xiexin Decoction (Yu et al., 2018), combination of Nutraceuticals (Rozza et al., 2009) and Altilix supplement containing chlorogenic acid and luteolin (Castellino et al., 2019).

**TABLE 5:** Summary of meta-analysis of polyherbal combinations used in Clinical studies in patients with MetS according to SPIDER model, concentration, quality control and chemical classifications reports.

	S	P	I	D	E		R	Other targets	References	Concentration	Quality control reported	Chemical analysis reported
S. No	Sample (size)	Population	Intervention/Phenomenon of interest	Study Design	Evaluation [MetS parameters assessed out of 5]	Evaluation Outcome (Parameters met)	Research Type (quantitative/qualitative)					
1	100 (50 control, 50 treatment)	Subjects with MetS complicated with MAU	Yiqi Huazhuo Gushen herbal formula (*Optis chinensis, Pollen typhae*, the rhizome of oriental water plantain, Mung bean peel, *Serissa serissoides*, Radix Aconiti *lateralis praeparata*)+ valsartan	Double-blinded and placebo-controlled	**5/5:** BMI, FPG, 2hPG, HbA1c, (HOMA-IR), SBP and DBP, MABP, TC, TG, LDL, HDL	**4/5:** reduced BMI, WHR, SBP, MAP, FPG, 2hPPG, HbA1c, reduce TG, increased HDL, LDL-c	Quantitative	Reductions in MAP, UACR, 24hTP and urinary β2 microglobulin	Tian-zhan et al. (2019)	Yes	No	No
2	60 (treatment = 30; control group = 30)	Subjects with MetS	Yiqi Huaju Qingli Formula with western medicine: Radix Astragali, Rhizoma Coptidis, *Pollen Typhae*, Artemisiae Rhizoma Alismatis, Testa Vignae Radiatae, *Serissa Japonica*, and Radix Aconiti *Lateralis Preparata*	Randomized placebo-controlled	**5/5:** BMI, WC, WHR, FPG, 2-hPPG, HbA1c, homeostasis model assessment for insulin resistance (HOMA-IR), TC, LDL, TG, HDL, BP	**4/5:** decreased BMI, WC, WHR, FPG, 2-hPPG, HbA1c, TG, increased HDL	Quantitative	reduced Urinary MA, UACR	Wang et al. (2013)	Yes	No	No
3	75 (Sesame+ vitamin E = 25, Sesame = 25; Sunflower oil = 25)	Subjects with MetS (aged 30–70 years)	Sesame oil and vitamin E	Randomized, single-blind controlled	**4/5** = dietary intake, BP, FBG, serum insulin, TC, TG, HDL	**4/5** = reduced TC, TG, FBG, HOMA-IR, SBP, DBP. increased HDL-c	Quantitative	MDA, Hs-CRP,	Farajbakhsh et al. (2019)	Yes	No but it was recruited from company	No
4	250 (63 per group; 4 groups	Subjects with MetS	*Curcuma longa* and *Nigella sativa*	Double blind randomized controlled	**5/5:** BMI, BF%, WC, HC, BP, TC, HDL-c LDL-c, TG, FBG	**3/5:** reduced BMI (weight, HC, BF%) FBG, TG, TC, LDL-c	Quantitative	CRP.	Amin et al. (2015b)	Yes	No	No
5	N = 116divided in 5 different groups	Type 2 diabetic subjects with MetS	Diabegon, (*Momordica charantia*, *Swertia chirata, Gymnema sylvestre, Trigonella foenumgraecum, Plumbago zeylanica, Eugena jambolana, Aegle marmelos, Terminalia chebula, Terminelia balerica, Emblica officinalis, Curcuma longa, Pterocarpus marsupium, Berberis aristata, Cytrullus culocynthis, Cyperus rotondus, Piper longum, root of Piper longum, Zingiber officinale, and* Asphaltum punjabinum	Double-blinded and placebo-controlled	**4/5:** BMI, FBG, TC, TG, LDL, HDL, VLDL	**3/5:** reduction in FBG, reduced TC, LDL, TG, increase HDL	Quantitative	reduction in uric acid, creatinine. Maintained LFTs (SGOT and SGPT)	Yadav et al. (2014)	Yes	No	No
6	21	Subjects with MetS (17–70 years)	Modified Lingguizhugan decoction (MLD)+ weekend fasting: (MLD = Poria, Ramulus Cinnamomi, Rhizoma *Atractylodis Macrocephalae*, and Radix Glycyrrhizae)	N/A	**5/5:** FPG, 2-h post-prandial blood glucose, fasting serum insulin (FINS), BP, BMI, WC, HOMA-IR, TG, TC, LDL-C, HDL-C	**3/5:** reduced FPG, HOMA-IR, PG, SBP, DBP, BMI, WC, LDL-C, decreased significantly	Quantitative		Yang et al. (2014b)	Yes	No but Pharmaceutical company provided it	No
7	450 (treatment = 225, Metformin = 225)	Type 2 diabetes	Dahuang Huanglian Xiexin Decoction (JTTZ): Aloe vera, *Coptis chinensis*, Rhizoma *Anemarrhenae*, red yeast rice, *Momordica charantia*, *Salvia miltiorrhiza*, *Schisandra chinensis*, and dried ginger	Positive-Controlled, Open-label	**3/5:** BMI, weight, WC, HC HbA1c, Total cholesterol, TG, FPG, 2 h PG, HOMA-IR, (HOMA-β), TC, LDLC	**3/5:** decreased HbA1c, FPG levels, TG and LDL-C levels, BMI, WC, HC	Quantitative		Yu et al. (2018)	No. established formula. Dose and duration given	Yes	Yes
8	30 (placebo = 15; treatment = 15)	Subjects with MetS	Nutraceuticals (Armolipid Prev, Rottapharm, Monza, Italy) + dietary intervention	Randomized, controlled, double-blind, parallel-group, single-centre	**5/5: B**MI, FBG, TG, HDL, SBP and DBP, TC, LDL	**3/5:** Reduce SBP and DBP, TG, LDL-C, TC, Increase HDL. MetS prevalence reduced from 15 to 5	Quantitative	N/A	Rozza et al. (2009)	registered drug so concentration may be in fixed preparation. Authors have not mentioned	No	No
9	100 (treatment = 50; placebo = 50	Subjects with MetS	Altilix® Supplement Containing Chlorogenic Acid and Luteolin	Randomized, Double-Blind	**4/5:** Body weight and BMI, FBG, HbA1c, Insulin resistance, pancreatic b cell function (HOMA-IR), TC, TG, LDL-C, HDL	**3/5:** Weight and BMI, improved Glycemic variables (HbA1c, HOMA-IR, and HOMA-β), reduced TC, TG, and LDL-C)	Quantitative	ALT, AST, GGT and AST/ALT ratio improved FLI, FMD, and cIMT improved, ghrelin levels reduced	Castellino et al. (2019)	No (prepared supplement-registered)	No	No
10	117 (treatment = 59; placebo = 58)	subjects with MetS	Curcuminoids (95% curcuminoids, of which at least 70% is curcumin) + piperine to enhance bioavailability	Randomized double-blind placebo-controlled	**2/5:** weight and BP	**2/5 =** reduction in Weight, height, SBP, DBP,	Quantitative	SOD, MDA, hs-CRP,	Panahi et al. (2015)	Patented ratio is mentioned but exact concentration not given	No	No
11	100 (placebo = 50; treatment = 50	Subjects with MetS	Curcuminoids (95% curcuminoids, of which at least 70% is curcumin) + piperine to enhance bioavailability	Randomized double-blind placebo-controlled parallel-group	**2/5:** TC, LDL-C, HDL-C, TG, LDL, lipoprotein and non-HDL-C	**2/5:** Reduced TG, elevated HDL-c, reduced TC, LDL-C, non-HDL-C	Quantitative		Panahi et al. (2014)	1000 mg curcuminoids per day with 10 mg piperine	No	No
12	50 (placebo = 26; treatment = 24)	Subjects with MetS	Red yeast rice and olive extract	Double blind placebo controlled randomized	**5/5**	**2/5**	Quantitative	CK elevation, ApoA1, ApoB, HbA1c and oxLDL	Verhoeven et al. (2015)	commercially available food supplement	Yes	Yes
13	30 healthy males; 45 obese divided into two groups	Centrally Obese men	Yiqi Sanju Formula	Randomized controlled	**2/5** = Insulin Resistance, BMI	**2/5 =** HOMA-IR and BMI reduced	Quantitative	high levels of CRP, FFA and PAI, t-PA was low	He et al. (2007)			
14	106 (treatment = 54; placebo = 52)	Adult subjects with MetS	Red yeast rice, bitter gourd, chlorella, soy protein, and licorice	double-blinded study	**5/5 =** BMI, BP, FBG, OGTT, TC, TGs, HDL, LDL	**2/5 =** reduced TG, BP, TC, LDL-c	Quantitative	No changes in LFT (ALT, AST, ALK-P) and renal functions test (serum creatinine, urea nitrogen, uric acid)	Lee et al. (2012)	Yes	Not mentioned but manufactured	No
15	100 (placebo = 46; treatment = 46)	subjects with MetS	Keishibukuryogan: Cinnamomi Cortex, Paeoniae Radix, Moutan Cortex, Persicae Semen, and Hoelen	controlled clinical trial with crossover design. Open labelled study; Quasi randomized	**5/5 =** BMI, HDL, LDL, FBG, TG, BP	**0/5**	Quantitative	L RHI increased, serum NEFA, MDA, and soluble vCAM1 decreased	Nagata et al. (2012)	Yes	No	No

Abbreviations: 24hTP, 24 h total urinary protein; 2hPPG, 2 h post prandial glucose; AST, aspartate aminotransferase; ALT, alanine transaminase; ALP, alkaline phosphatase; BP, blood pressure; BMI, body mass index; CDKAL = CDK5 Regulatory Subunit Associated Protein 1 Like 1); CETP, cholesteryl ester transfer protein; CRP, C-reactive protein; FBG: fasting blood glucose; FFA, free fatty acid; GLUT-4, glucose transporter 4; GGT, glutamyl-transferase; HC, hip circumference; HDL-C, high density lipoproteins; HFD, high fat diet; HOMA-IR, homeostatic model assessment for insulin resistance; ICAM-1, intercellular adhesion molecules; IRS-1, Insulin receptor substrate 1; KKAy, cross between diabetic KK and lethal yellow; LDL, low density lipoprotein; Monocyte chemoattractant protein-1; MAU/*MA*, microalbuminuria; MDA, malondialdehyde; NAFA, non-esterified fatty acids; NF-kB, Nuclear Factor kappa-light-chain-enhancer of activated B cells; PI3K phosphoinositide 3-kinase SREBP, sterol regulatory element-binding transcription factor; PPAR-γ/a, Peroxisome proliferator-activated receptor gamma/alpha; SD Rats, Sprague Dawley rats; SGOT, Serum glutamic oxaloacetic transaminase; SGPT, Serum glutamic pyruvic transaminase; SOD, superoxide dismutase; TC, total cholesterol; TG, triglycerides; TNF, Tumor necrosis factor; vLDL, very low density lipoprotein; UACR, urea creatinine albumin ratio; vCAM-I, Vascular cell adhesion molecule 1; WC, waist circumference; WHR, waist hip ratio; WKy, Wistar Kyoto.

**TABLE 6:** Qualitative scoring of clinical trials.

Code: Yes = 1; No = 2; 3 = can't say											Link
References	Addressed clearly focused question	Subjects to treatment groups randomised	An adequate concealment method	Subjects and investigators “blind”	The treatment and control groups are similar at the start of the trial	The only difference between groups is the treatment under investigation	All relevant outcomes are measured in a standard, valid and reliable way	Dropped out before study completion	All the subjects are analysed in the groups to which they were randomly allocated	Where the study is carried out at more than one site, results are comparable for all sites	
Wang et al., 2019 (Tian-zhan et al., 2019)	1	1	1	1	1	1	1	0	1	2	https://www.ajol.info/index.php/tjpr/article/view/183342
Wang et al. (2013)	1	1	1	1	1	1	1	3	1	2	https://pubmed.ncbi.nlm.nih.gov/23743161/
Mazloomi et al., 2019 (Farajbakhsh et al., 2019)	1	1	1	1	1	1	1	5 (6%)	1	2	https://pubmed.ncbi.nlm.nih.gov/31089253/
Amin et al. (2015b)	1	1	1	2	1	1	1	rate was low	1	2	https://www.sciencedirect.com/science/article/abs/pii/S0965229915000096?via%3Dihub
Yadav et al. (2014)	1	3	3	3	2	2	1	3	1	2	https://www.ncbi.nlm.nih.gov/pmc/articles/PMC4202628/
Yang et al. (2014b)	2	2	2	3	2	1	1	3	1	2	https://pubmed.ncbi.nlm.nih.gov/25102690/
Yu et al. (2018)	1	1	1	2	1	1	1	10/225 (treatment); 26/225 (metformin group)	1	2	https://www.hindawi.com/journals/ije/2018/9519231/
Rozza et al. (2009)	1	1	3	1	1	1	1	0	1	2	https://pubmed.ncbi.nlm.nih.gov/23334909/
Castellino et al. (2019)	1	1	1	1	1	1	1	0	1	2	https://www.ncbi.nlm.nih.gov/pmc/articles/PMC6893885/
Panahi et al. (2015)	1	1	1	1	1	1	1	curcuminoids (9/59) placebo (8/58)	1	2	panahi2015.pdfhttps://pubmed.ncbi.nlm.nih.gov/25618800/
Panahi et al. (2014)	1	1	1	1	1	1	1	curcuminoids (9/59) placebo (8/58)	1	2	https://pubmed.ncbi.nlm.nih.gov/25440375/
Verhoeven et al. (2015)	1	1	1	1	1	1	1	1/25 from intervention group	1	2	https://bmccomplementmedtherapies.biomedcentral.com/articles/10.1186/s12906-015-0576-9
Wang et al., 2007 (He et al., 2007)	1	1	1	1	1	1	1	N/A	1	2	http://www.jcimjournal.com/EN/10.3736/jcim20070307
Lee et al. (2012)	1	1	1	1	1	1	1	2/54 (treatment) and 8/52 (placebo)	1	2	https://pubmed.ncbi.nlm.nih.gov/22348456/
Nagata et al. (2012)	1	**1**	2	2	2	1	1	19/46 in Group A; 24/46 Group B	1	2	https:// www.hindawi.com/journals/ecam/2012/359282/

## Discussion

MetS is a cluster of metabolic abnormalities that appear as a pre-diseased state and predisposes to CVD risk even before overt disease such as diabetes or hypertension develops. Catering those risk factors at this stage could prevent incidence of CVD. Hence, clinicians need to target multiple risk factors simultaneously. As the incidence of MetS is rising, there is a need to identify therapeutic modalities that could address multiple disease targets, offer better compliance, and reduce risk of adverse effects (Reilly and Rader, 2003; Keith et al., 2005). Polyherbal formulations could mutually enhance pharmacological synergy on the targeted disease and often exhibit pharmacological and therapeutic superiority in comparison to isolated single constituents.

The current review focuses on studies published from 2005–2020, reporting the efficacy of polyherbal therapies in MetS. This is attributed to either the action of bioactive ingredients from different herbs on the same molecular target forming a multiple-drug-one-target model (additive effect) and/or the functionally diverse targets but with potentially clinically relevant associations forming a multiple-drug-multiple-target-one-disease (synergistic effect) (Lu et al., 2012; Wang et al., 2012). In the current review, we identified 25 animal based studies in which polyherbal formulations were used in animal models of Mets. We categorised them as good and not very good, based on the modulation of MetS parameters. Studies which were able to modulate 4-5 parameters were considered as very effective, whereas studies that modulated three or less than 3 parameters were marked as not so good. This, however, does not reflect on the quality of review. For the quality of review, we devised an 8-question checklist and marked one point for meeting the criteria and 0 for no meeting the criteria. The overall score was 8.

From the effect point of view, different combinations were identified as very effective in animal based studies. They included combination of *Curcuma longa*, *Salacia reticulate*, *Gymnema sylvestre*, *Emblica officinalis*, *Terminalia chebula* (Thota et al., 2014), *Glycyrrhizae uralensis Fischer*, *Rheum undulatum Linne*, *Prunus persica Linne*, *Cinnamomum cassia Presl* and *Natrii Sulfas* (Sung et al., 2014), *Rhizoma coptidis*, *Radix scutellariae*, *Cortex phellodendri* and *Fructus gardeniae* (Li et al., 2013), Red ginseng and *Polygoni Multiflori Radix* (Kho et al., 2016) and modified lingguizhugan decoction (Yao et al., 2017a). These combinations modulated all the five parameters of MetS including reduction in body weight/obesity, BP, TG, and fasting blood glucose (FBG) and increase in HDL. Additionally, combination of soybean meal and probiotics (*Bifidobacterium longum*) (Mounts et al., 2015), Fu Fang Zhen Zhu Tiao Zhi formula (Hu et al., 2014), *Curcuma Longa* and *Nigella Sativa* (Amin et al., 2015a) and mixed extracts of *Artemisia iwayomogi* and *Curcuma longa* (Lee et al., 2015a) improved 4/5 MetS parameters and can be further considered for clinical trials.

These studies however exhibited certain limitations. For example, Lee et al. (2015a), comprehensively studied effect of *Artemisia iwayomogi* and *Curcuma longa* extract on metabolic markers along with fine mechanistic details but did nto use positive controls in their study. Similarly, Yao et al. (2017a) did not use positive controls in their study when studying effect of modified Lingguizhugan decoction (MLD) and only selected one dose for intervention. Hence, dose dependent effect couldn’t be assessed. Besides, they did not study the effect mediated by MLD alone and only showed results of MLD with dietary restriction and exercise; additional group of MLD should have been added for confidently claiming the effect of MLD in the study. Amin et al., presented their findings comprehensively about use of combined *Curcuma longa* and *Nigella sativa* in MetS models but despite of mention of measuring body weight fortnightly, there were no results about effect on body weight (Amin et al., 2015a).

Some studies showed reduced effect on Met S parameters, but their focus was more on mechanistic details. For instance, study by Gao et al. (2015) on effect of Erchen decoction (ECD) exhibited effect on 3 parameters of MetS including FBG, TG and body weight and abdominal circumference. One of the appreciable aspects of this study is that the researchers reported abdominal circumference and body weight simultaneously. Limited animal studies consider abdominal circumference, which is the actual predictor of MetS. Additionally, molecular mechanisms of ECD on diabetic parameters have been elaborated at genetic level, where expression of CDK5 regulatory subunit associated protein 1 like 1 (CDAK1) has been shown and correlated with improved islet cell function. Since this preparation did not have effect on LDL and HDL, combining it with antidyslipidemic herb, such as *Curcuma longa* and/or *Nigella sativa* coupled with low dose of ECD may be a good combination for future studies. Like this, extracts of *Salvia miltiorrhiza* and *Gardenia jasminoides* (Tan et al., 2013), showed effect on 3 parameters of Met S, but gave an elaborate mechanism for their antiobesity effect including enhanced leptin expression. Amongst the studies reported in this review, limited studies assessed BP (Thota et al., 2014; Amin et al., 2015a); whereas, most of them did not assess blood pressure in their models, and therefore the studies which have either met 3 or 4 out of 5 parameters of MetS are majorly the ones which did not assess BP in their animal models (Mounts et al., 2015). One of the reasons for this could be that BP monitoring in animals is technically challenging, and assessing it for number of animals, which usually are 40–50 altogether, is highly tedious and time consuming.

The other part of our review focussed on clinical trials in the last 15 years which used polyherbal formulations for the management of MetS. Amongst the combinations reviewed the most effective considered were the ones which met maximum MetS parameters. The maximum parameters modified were 4 out of 5 by 3 combinations including Yiqi Huazhuo Gushen herbal formula (Tian-zhan et al., 2019), Yiqi Huaju Qingli Formula (Wang et al., 2013), and Sesame oil and vitamin E combination (Farajbakhsh et al., 2019). However, these studies were assessed for short period of time ranging from 8 to 12 weeks, which may be helpful in determining the acute effect but not long-term effect and side-effects.

From this perspective a study by Yadav et al. (2014) is worth mentioning who studied the effects of herbal combination “Diabegon” till 1.5 years and monitored the effect on liver and kidney parameters, which showed no toxic effects on these organs. In fact, the combination reduced uric acid and effectively reduced FBG, TG and increased HDL, although BP was not monitored. Another worthy study in this regard was controlled clinical trial which used Keishibukuryogan, a traditional Japanese (Kampo) formula (Nagata et al., 2012) in MetS patients in a cross over design. Although, it did not reduce any MetS parameters, its main outcome was improvement in endothelial function which has a preventive role towards atherosclerosis. Such study designs should be adopted for formulas which have shown promising results in small scale studies.

Some studies design was flawed and therefore the effects could not be validated. For instance, study by Yang et al. (2014a) on MLD along with weekend fasting tested the combination on MetS patients but no comparative control was used. We could not determine whether the effect was due to MLD or weekend fasting. Aims of the study were also not clearly written in the write-up. Similarly, a combination of nutraceuticals with dietary interventions very efficiently reflected the improvement in MetS parameters to an extent that the patients no longer fulfilled the MetS criteria after treatment (10/15) (Rozza et al., 2009). Nevertheless, with such a small sample size, the magnitude of impact could not be extrapolated and needs to be studied further. Some clinical studies assessed only limited parameters of MetS and therefore in terms of effectiveness those combinations are considered as not so good. Nevertheless, that’s not completely true, because the authors did not measure the remaining parameters (He et al., 2007; Panahi et al., 2014; Panahi et al., 2015). Reason for this could be that the main objective of those studies was to explore additional mechanisms of MetS. For instance, Panahi et al., (Panahi et al., 2015) report curcuminoids to reduce 2 out of 5 MetS parameters because they assessed only BP and BMI. Their main finding was anti-inflammatory and antioxidant activities, whereas antidyslipidemic effect was reported in their preceding study (Panahi et al., 2014).

The current review has certain limitations. One of the factors to be considered for future reviews should be to differentiate the polyherbal combinations according to different ethnicities and cultures in which the herb is famously used such as Asian, Chinese and Japanese traditional medicine. The current review can be used by researchers for idnetifying different polyherbal combinations by considering which herbs could simultaneously target many or all risk factors for MetS. For future studies some known anti-obesity and/or antihypertensive herbs shall be considered as an add-on with those polyherbal combinations that predominantly exhibited anti-hyperglycaemic and anti-dyslipidemic effect, to be able to manage multiple MetS parameters simultaneously. This is one of the advantages of such reviews that researchers could identify the missing targets and add herb accordingly for future studies.

## Data Availability

The original contributions presented in the study are included in the article/Supplementary Material, further inquiries can be directed to the corresponding authors.
