# Supplementary material for: Systematic Review of Polyherbal Combinations Used in Metabolic Syndrome
Source: Front Pharmacol. 2021 Oct 7;12:752926. doi: 10.3389/fphar.2021.752926 (PMC8529216; doi:10.3389/fphar.2021.752926)
Supplement: Supplementary file 1 [file Table1.docx]

**Supplementary Table 1: Summary of meta-analysis of polyherbal combinations used in clinical studies on efficacy of herbal combinations in patients with MetS according to SPICE model**

|  | **S** | **P** | **I** | **C** | **E** | |  |  |
| --- | --- | --- | --- | --- | --- | --- | --- | --- |
| **S.NO** | **Settings** | **Population** | **Intervention/Phenomenon of interest** | **Comparison of treatment with placebo/Controls** | **Evaluation [Assessed MetS parameters out of 5]** | **Evaluation Outcome** | **References** |  |
| 1 | Department of the Traditional Chinese Medicine, Shanghai Jiao Tong University Affiliated Sixth People’s Hospital | subjects with MetS complicated with MU | Yiqi Huazhuo Gushen herbal formula (*Optis chinensis*, *Pollen typhae*, the rhizome of oriental water plantain, Mung bean peel, *Serissa serissoides*, Radix coniti lateralis praeparata) + valsartan | positive control given Valsartan | **Parameter assessed:** BMI, FPG, TC, TG, LDL-c, SBP and DBP, MABP, postprandial 2 h blood glucose (2hPG), HbA1c, (HOMA-IR), TC, TG, LDL, HDL | **Parameters met: 4/5** reduced BMI, WHR, SBP, MAP, FPG, 2hPPG, HbA1c, reduce TG, increased HDL, LDL-c | ([43](#_ENREF_43)) |  |
| 2 | Hospital Setting | subjects with MetS | Yiqi Huaju Qingli Formula with western medicine: Radix Astragali, Rhizoma Coptidis, Pollen Typhae, Artemisiae Rhizoma Alismatis, Testa Vignae Radiatae, Serissa  japonica, and Radix Aconiti Lateralis preparata. | positive control given western medicine | **Parameters assessed: 5/5**  BMI, WC,WHR, FPG, 2-hPPG, HbA1c, HOMA-IR, TC, LDL, TG, HDL, BP | **Parameters met: 4/5** decreased BMI, WC,WHR,FPG, 2-hPPG, HbA1c, TG, increased HDL | ([44](#_ENREF_44)) |  |
| 3 | Shiraz Heart Center Outpatient Clinic | subjects with MetS | Sesame oil and vitamin E | sunflower oil and vitamin E | **Parameters assessed: 5/5**  dietary intake, BP, FBG, serum insulin,TC, TG,HDL | **Parameters met:** 4/5 reduced TC, TG, FBG, HOMA-IR, increase HDL-c, SBP, DBP | ([45](#_ENREF_45)) |  |
| 4 | Hijrat colony, Karachi, Pakistan. | subjects with MetS | *Curcuma longa* and *Nigella sativa* | Met S patients given placebo matched with drug capsules | **Parameters assessed: 5/5**  BMI, BF%, WC, HC, BP, TC, HDL-c LDL-c, TG, FBG | **Parameters met:** 3/5 reduced BMI (weight, HC, BF%) FBG, TG  TC, LDL-c | ([46](#_ENREF_46)) |  |
| 5 | weekend diabetic clinic run in the school of studies in Biotechnology at Jiwaji University Gwalior. | Type 2 diabetic subjects with MetS | Diabegon, (Momordica charantia, Swertia chirata, Gymnema sylvestre, Trigonella foenumgraecum, Plumbago zeylanica, Eugena jambolana, Aegle marmelos, Terminalia chebula, Terminelia balerica, Emblica officinalis, Curcuma longa, Pterocarpus marsupium, Berberis aristata, Cytrullus culocynthis, Cyperus rotondus, Piper longum, root of Piper longum, Zingiber officinale, and Asphaltum punjabinum | No controls, different type of NIDDM groups with varying FBS and age. Compared amongst each other. | **Parameters assessed: 4/5**  BMI, FBG, TC, TG, LDL, HDL, VLDL, | **Parameters met: 3/5** reduction in FBG, reduced TC, LDL, TG, increase HDL, | ([47](#_ENREF_47)) |  |
| 6 | Affiliated Hospital of Sun Yat-Sen University. | Subjects with MetS (17-70 years) | modified Lingguizhugan decoction (MLD)+ weekend fasting: (MLD = Poria, Ramulus Cinnamomi, Rhizoma Atractylodis Macrocephalae, and Radix Glycyrrhizae) | No controls | **Parameters assessed: 5/5** FPG, 2-h post-prandial blood glucose, FINS, BP, BMI, WC, HOMA-IR, TG, TC, LDL-C, HDL-C | **Parameters met: 3/5**  reduced FPG, HOMA-IR, PG, SBP, DBP, BMI, WC, LDL-C decreased | ([48](#_ENREF_48)) |  |
| 7 | Guang’anmen Hospital of the China Academy of Chinese Medical Sciences | Type 2 diabetes | Dahuang Huanglian Xiexin Decoction ( JTTZ): Aloe vera, Coptis chinensis, Rhizoma Anemarrhenae,red yeast rice, Momordica charantia, Salvia miltiorrhiza, Schisandra chinensis, and dried ginger. | positive control metformin 0.25 mg tid | **Parameters assessed: 3/5** BMI, weight, WC, HC HbA1c,TC, TG, FPG, 2 h PG, HOMA-IR, HOMA-β), TC, LDLC | **Parameters met: 3/5** decreased HbA1c ,FPG levels ,TG and LDL-C levels , BMI, WC and HC | ([49](#_ENREF_49)) |  |
| 8 | Hospital Setting | subjects with MetS | Nutraceuticals ((Armolipid Prev , Rottapharm, Monza, Italy)+ dietary intervention | placebo + dietary intervention | **Parameters assessed: 5/5** BMI, FBG, TG, HDL, SBP & DBP, TC, LDL | **Parameters met: 3/5** Reduce SBP & DBP, TG, LDL-C, TC, Increase HDL. MetS prevalence reduced from 15 to 5 | ([50](#_ENREF_50)) |  |
| 9 | Unit of Diabetes and Cardiovascular Prevention, University Hospital of Palermo, Italy | subjects with MetS | Altilix® Supplement Containing Chlorogenic Acid and Luteolin | MetS patients given placebo | **Parameters assessed:4/5**  body weight and BMI, FBG, HbA1c, HOMA-IR, TC, TG, LDL-C, HDL | **Parameters met: 3/5** improved Weight and BMI, HbA1c, HOMA-IR, and HOMA-β, reduced TC, TG, and LDL-C | ([51](#_ENREF_51)) |  |
| 10 | Cardiology or Endocrinology Clinics of the Baqiyatallah Hospital (Tehran, Iran) | subjects with MetS | curcuminoids—(piperine was added to enhance Bioavailability) (95% curcuminoids, of which at least 70% is curcumin) | Met S patients given placebo matched with drug capsules containing d lactose (inert material) plus 5 mg of Bioperine (contains 95% piperine) | **Parameters assessed: 2/5** | **Parameters met: 2/5** reduction in Weight, height, SBP, DBP, | ([75](#_ENREF_75)) |  |
| 11 | Cardiology or Endocrinology Clinics of the Baqiyatallah Hospital (Tehran, Iran) | subjects with MetS | curcuminoids—(piperine was added to enhance Bioavailability) (95% curcuminoids, of which at least 70% is curcumin) | Met S patients given placebo matched with drug capsules containing d lactose (inert material) plus 5 mg of Bioperine (contains 95% piperine) | **Parameters assessed: 2/5** TC, LDL-C, HDL-C, TG, sdLDL, lipoprotein and non-HDL-C | **Parameters+G9 met: 2/5** Reduced TG, elevated HDL-c, reduced TC, LDL-C, non-HDL-C | ([76](#_ENREF_76)) |  |
| 12 | Not mentioned; | subjects with MetS | Red yeast rice and olive extract | Met S patients given placebo | **Parameters assessed: 5/5** BMI, SBP, DBP, FBG, Tc, LDL & HDL, TG | **Parameters met: 2/5** reduced TG, Apo B, SBP and DBP, TC, LDL, | ([77](#_ENREF_77)) |  |
| 13 | Hospital setting | centrally Obese men | Yiqi Sanju Formula | 30 healthy male as negative control, 23 obese male given placebo | **Parameters assessed:** 2/5 Insulin Resistance, BMI; | **Parameters met:** 2/5 HOMA-IR and BMI reduced | ([78](#_ENREF_78)) |  |
| 14 | outpatient clinic Division of Endocrinology and Metabolism in Taichung Veterans General Hospital, Taiwan. | Subjects with MetS | Red yeast rice, bitter gourd, chlorella, soy protein, and licorice | placebo given to MetS subjects | **Parameters assessed: 5/5**  BMI, BP, FBG, OGTT, TC, TGs, HDL, LDL | **Parameters met: 2/5** reduced TG, BP, TC, LDL-c, | ([79](#_ENREF_79)) |  |
| 15 | Department of Japanese Oriental Medicine, Toyama University Hospital | subjects with MetS | Keishibukuryogan: Cinnamomi Cortex, Paeoniae Radix, Moutan Cortex, Persicae Semen, and Hoelen. | placebo control | **Parameters met: 5/5** BMI,HDL,LDL, FBG, TG, BP | **Parameters assessed: 0/5** | ([80](#_ENREF_80)) |  |
| **Abbreviations:** ratio of urinary albumin to creatinine = (UACR), Microalbuminuria = MA, 24- h total volume of urinary protein (24hTP), SEFA = nonesterified fatty acid, vCAM1 = vascular cell adhesion molecule 1; Fatty liver index = FLI; FMD = flow mediated dilation; cIMT; natural logarithmic-scaled reactive hyperemia index = L RHI; NEFA = serum nonesterified fatty acid; MDA = malondialdehyde,; vCAM-1 = vascular cell adhesion molecule 1; HOMA-IR = homeostasis model assessment for insulin resistance ( insulin resistance index), HOMA-β = β cell function index; FINS = fasting serum insulin;; TC = total cholesterol, HDL-c = high density lipoprotein cholesterol, LDL-c = low density lipoprotein cholesterol, TG = triglyceride, FPG = fasting plasma glucose, FBG = fasting blood glucose, BP = blood pressure, SBP = systolic blood pressure, DBP = diastolic blood pressure | | | | | | | | |
